# Potential Therapeutic and Prognostic Values of LSM Family Genes in Breast Cancer

**DOI:** 10.3390/cancers13194902

**Published:** 2021-09-29

**Authors:** Hoang Dang Khoa Ta, Wei-Jan Wang, Nam Nhut Phan, Nu Thuy An Ton, Gangga Anuraga, Su-Chi Ku, Yung-Fu Wu, Chih-Yang Wang, Kuen-Haur Lee

**Affiliations:** 1 PhD Program for Cancer Molecular Biology and Drug Discovery, College of Medical Science and Technology, Taipei Medical University and Academia Sinica, Taipei 11031, Taiwan; d621109004@tmu.edu.tw (H.D.K.T.); g.anuraga@unipasby.ac.id (G.A.); 2 Graduate Institute of Cancer Biology and Drug Discovery, College of Medical Science and Technology, Taipei Medical University, Taipei 11031, Taiwan; b101104152@tmu.edu.tw; 3 Department of Biological Science and Technology, Research Center for Cancer Biology, China Medical University, Taichung 40402, Taiwan; cvcsky@cmu.edu.tw; 4 Institute for Environmental Science, Nguyen Tat Thanh University, Ho Chi Minh City 700000, Vietnam; pnnam@ntt.edu.vn; 5 NTT Institute of Hi-Technology, Nguyen Tat Thanh University, Ho Chi Minh City 700000, Vietnam; tntan@ntt.edu.vn; 6 Department of Statistics, Faculty of Science and Technology, Universitas PGRI Adi Buana, Surabaya 60234, Indonesia; 7 National Defense Medical Center, Department of Medical Research, School of Medicine, Tri-Service General Hospital, Taipei 11490, Taiwan; qrince@yahoo.com.tw; 8 Cancer Center, Wan Fang Hospital, Taipei Medical University, Taipei 11031, Taiwan

**Keywords:** biomarker, breast cancer, LSM1, LSM2, LSM3, LSM4, LSM7, LSM14B

## Abstract

**Simple Summary:**

The roles of “like-Smith” (LSM) proteins in breast cancer development and their clinical relevance remain unclear. In this study, multiple analyses based on 3593 patients with breast cancer and their mRNA expression values were utilized to investigate the clinical relevance of LSM family genes, including cancer aggressiveness, immune cell infiltration, prognostic outcomes, and related signaling pathways. We revealed that LSM4 had higher expression levels in breast tumor and breast cancer sub-types than in normal samples, and was associated with poor survival outcomes. Interestingly, infiltration levels of most immune cell types, including cluster of differentiation for positive CD4^+^ T cells, CD8^+^ T cells, T-cell follicular helpers, and myeloid-derived suppressor cells were positively correlated with LSM4 expression in several subclasses of breast cancer (basal, human epidermal growth factor receptor 2 (HER2), luminal A, and luminal B).

**Abstract:**

In recent decades, breast cancer (BRCA) has become one of the most common diseases worldwide. Understanding crucial genes and their signaling pathways remain an enormous challenge in evaluating the prognosis and possible therapeutics. The “Like-Smith” (LSM) family is known as protein-coding genes, and its member play pivotal roles in the progression of several malignancies, although their roles in BRCA are less clear. To discover biological processes associated with LSM family genes in BRCA development, high-throughput techniques were applied to clarify expression levels of LSMs in The Cancer Genome Atlas (TCGA)-BRCA dataset, which was integrated with the cBioPortal database. Furthermore, we investigated prognostic values of LSM family genes in BCRA patients using the Kaplan–Meier database. Among genes of this family, LSM4 expression levels were highly associated with poor prognostic outcomes with a hazard ratio of 1.35 (95% confidence interval 1.21–1.51, p for trend = 3.4 × 10^−7^). MetaCore and GlueGo analyses were also conducted to examine transcript expression signatures of LSM family members and their coexpressed genes, together with their associated signaling pathways, such as “Cell cycle role of APC in cell cycle regulation” and “Immune response IL-15 signaling via MAPK and PI3K cascade” in BRCA. Results showed that LSM family members, specifically LSM4, were significantly correlated with oncogenesis in BRCA patients. In summary, our results suggested that LSM4 could be a prospective prognosticator of BRCA.

## 1. Introduction

According to Global Cancer statistics, the estimated 2.3 million confirmed cases of breast cancer (BRCA) in 2020 elevated it to become the most prominent malignant cancer worldwide [1]. BRCA accounts for one out of four cancers and kills one out of six women, making it the most prevalent cancer among women worldwide. In developing countries, the rise in BRCA is further aggravated by economic conditions and leads to several social complications [2,3,4,5,6]. Therefore, it is imperative to build a supportive infrastructure to promote cancer prevention and treatments [7,8,9,10,11]. Although great efforts to discover new therapeutics have been greatly made, the survival rates of patients with BRCA remained relatively low [12,13,14,15]. Past researches have found that cancer can be detected at an early stage by screening the differentially expressed genes (DEGs) as prognostic biomarkers in associated with poor survival rate in cancer patients to design target drugs [16,17,18]. Fortunately, with the rapid development in both computing abilities and big data resources, large publicly available datasets have been constructed for academic research and even for commercialization [19].

Smith-like (LSM) proteins are known as a family of RNA-binding proteins that appear in essentially all cellular organisms [20]. First discovered in a patient with systemic lupus erythematosus, these so-called Sm proteins are antigens targeted by anti-Sm antibodies [21].

The LSM family has 13 members (LSM1~LSM14B) which were strongly associated with tumorigenesis and metastasis of several tumor types. In particular, the LSM1 protein plays roles in the cellular conversion and progression of BRCA, mesotheliomas, and lung cancer [22,23,24], and in the metabolism of RNA [25]. LSM3 was reported to be significantly associated with Alzheimer’s disease [26], while LSM8 has a strong relationship with the development of Hashimoto’s thyroiditis [27].

However, the biological functions of each member of the LSM family genes are not fully understood, especially with regard to the tumor microenvironment (TME) [28]. Multiple amounts of microarray and sequencing technologies have enhanced the ability of robust computational algorithms to rapidly analyze biomedical data rapidly [29,30,31]. In spite of this, challenges remain in imitating the human TME in vivo and in vitro. Employing genes expressions and appropriate algorithms is believed to help us understand the respective immune functions. In this study, we integrated several high throughput data and platforms to reveal insights into the molecular mechanisms of LSM family members and to clarify potential therapeutic targets for BRCA. Furthermore, we investigated the survival rate of BRCA patients based on LSM messenger (m)RNA transcription levels, using a Kaplan–Meier (KM) plot. Currently, the roles of LSM members in the development of any diseases are vague [32,33]; therefore, we attempted to predict the molecular functions and signaling pathway networks by applying MetaCore, a high-quality biological platform for multi-omics data.

Comprehensive methods can help reveal the roles of LSM family genes in BRCA growth, improve prognoses, and personalize effective treatments. We hypothesized that the LSM family, especially LSM4, might possess a novel role in tumor growth, and infiltration of immune cells may provide better predictions of survival rates in BRCA patients.

## 2. Materials and Methods

### 2.1. UALCAN

UALCAN (http://ualcan.path.uab.edu, accessed on 1 May 2021) is an inclusive puplic database using The Cancer Genome Atlas (TCGA) “Level 3” RNA-sequencing (RNA seq) and clinical data from more than 30 cancer types. This database includes expression values computed by the RSEM algorithm for 20,502 genes [34]. Transcripts per million (TPM) was used to evaluate whether the difference in gene expression levels between groups were statistically significant. This platform retrieved data from TCGA, including 114 normal samples and 1097 primary BRCA tumors. Our report utilized the mRNA levels of 13 LSM family genes in breast cancer and their correlations with clinicopathological parameters and tumor stages. Additionally, we also performed a comprehensive analysis of promoter DNA methylation level in both control group (*n* = 97) and tumor group (*n* = 793) base on TCGA dataset. The beta value showed the degree of DNA methylation ranging from 0 (unmethylated) to 1 (fully methylated). Student’s *t*-test was used, and a *p* < 0.05 was considered as statistically significant.

### 2.2. DNA Methylation

We used Methsurv (https://biit.cs.ut.ee/methsurv/, accessed on 1 May 2021) to create a heatmap of the various DNA methylated regions in order to evaluate the methylation level of a target gene [35]. Beta values were used to reflect DNA methylation levels (ranging from 0 to 1). M/ (M + U + 100) is used to calculate the beta value for each CpG site. M and U represent the methylated and unmethylated intensities, respectively.

### 2.3. Functional Enrichment Analysis

We obtained data from the METABRIC (*n* = 2509) and TCGA datasets (*n* = 1084) in the cBioPortal (https://www.cbioportal.org, accessed on 1 May 2021) database [36,37,38]. The aim of this analysis was to determine biological processes (BPs), disease biomarker networks, and breast neoplasm cell-cell signaling pathways using the MetaCore analysis (https://portal.genego.com, accessed on 1 May 2021). Furthermore, a gene ontology (GO) analysis was also implemented to describe genes and gene products from three categories: cellular components (CPs), molecular functions (MFs), and BPs by obtaining data from DOSE packages in R (vers. 4.0). Of note, we also performed a gene set enrichment analysis (GSEA, http://software.broadinstitute.org/gsea, accessed on 1 May 2021) to identify gene product activities in BRCA, with a dataset obtained from the METABRIC database. A q-value false discovery rate (FDR) and normalized enrichment score (NES) were calculated. A q-value of <0.25 was set as the boundary criterion as previously described, and an NES of >1.5 and a nominal *p* value of <0.05 were set as the thresholds [36,39,40,41,42,43,44,45,46].

### 2.4. Survival Analysis

We determined the correlations between LSMs mRNA expression levels and the survival of BRCA patients using the KM-plot database (https://kmplot.com, accessed on 1 May 2021). This online public database server is a robust platform for visualizing patients with several cancer types included in TCGA and METABRIC databases. Additionally, gene expression and survival data were taken from the Gene Expression Omnibus (GEO) and TCGA (HG-U133A 2.0, Affymetrix HG-U133A, and HG-U133 Plus 2.0 microarrays). Of note, this platform contains 22,277 genes on BRCA prognoses with microarray data from 1809 patients [47]. It was developed to assess the influence of target genes on the prognosis of patients with BRCA. Recurrence-free survival (RFS) for the LSM gene family was set as the default in the KM-plot database [47], including a survival curve, p log-rank value, and hazard ratios (HRs) with 95% confidence intervals (CIs), all of which were maintained in the plot. The horizontal axis (*x*-axis) showed the survival time in months, and the vertical axis (*y*-axis) showed the probability of survival.

### 2.5. Analysis of Protein Expression in Clinical Specimen

LSM family protein expressions were further calculated using the publicly available Human Protein Atlas (HPA) web database, which contains more than 10 million IHC images and 82,000 high-resolution immunofluorescences (IF) images of tissue microarrays. These microarrays, which contain sections from 46 normal human tissues and more than 20 types of human cancers, were labeled with antibodies against more than 11,000 human proteins. [48]. We obtained 1075 patient samples from the BRCA data resources, and evaluated protein expressions on IHC images in clinical samples. The staining is reported in terms of intensity, subcellular localization, and single-cell variability (SCV) for each cell line and antibody. Based on the laser power and detector gain parameters utilized for image capture in combination with the visual appearance of the image, the staining intensity is categorized as negative, weak, moderate, or strong. Protein expression score is determined by manually scoring immunohistochemical data for staining intensity (negative, weak, moderate, or strong) and proportion of stained cells (25 percent, 25–75 percent, or >75 percent). To automatically translate each intensity and fraction combination into a protein expression level score, the following formula is used: negative–not detected; weak < 25%–not detected; weak combined with either 25–75% or 75%–low; moderate < 25%–low; moderate combined with either 25–75% or 75%–medium; strong < 25%–medium, strong combined with either 25–75% or 75%–high.

### 2.6. TIMER Analysis

Using TIMER 2.0 (http://timer.comp-genomics.org/, accessed on 1 May 2021), we explored the infiltration levels of immune cells in 31 cancer types, and approximately 10,000 samples were obtained from the TCGA dataset. All TCGA tumor data were retrieved from GDAC (http://firebrowse.org/, accessed on 1 May 2021), and included somatic mutation cells, somatic copy number variations, transcriptome profiles, and clinical outcomes. Gene expression levels were expressed using log2[relative standard error of the mean (RSEM)]. To obtain expression levels in normal and cancer tissues, LSM genes under the DiffExp module with default settings were used. The “DiffExp” module allows for the investigation of differences in gene expression between normal and malignant tissues for any gene across all TCGA tumors. The “correlation” module presents expression scatterplots between two user-defined genes in a certain cancer type, along with Spearman correlation coefficients and statistical significance, and can be adjusted by tumor purity [49]. From default immune cells such as B cells, CD8^+^ T cells, macrophages, CD4^+^ T cells, neutrophils, and dendritic cells, we investigated relationships between highly expressed LSMs and the infiltration of inflammatory cells in several BRCA subtypes.

### 2.7. Differentially Expressed Genes (DEGs) Analysis

We attempted to validate the results using the Genomic Data Commons (GDC) database (https://portal.gdc.cancer.gov, accessed on 1 May 2021), which contains more than 50,000 raw sequencing data inputs, and various other data types. We first queried all of the genome data by applying TCGA Biolinks package in R (vers. 4.1.0), and then created a violin plot to compare gene expression levels between healthy tissues and tumor tissues. The Mann-Whitney test was applied to compare the two paired groups. A heatmap of expression levels of 1222 BRCA patients was also performed using the “DESeq2” package to illustrate differences between the two phenotypes: “normal” and “tumor”. We selected the top 10% of genes, ranked by the log2[fold change (FC) expression values], with |logFC| > 1.5 set as the threshold [50,51,52,53,54].

### 2.8. Statistical Analysis

We utilized TCGA Pan-Cancer Atlas, a dataset from cBioPortal (https://www.cbioportal.org, accessed on 1 May 2021), to obtain patient data and query the effects of the expressions of different LSM family members on overall survival (OS). For the survival analysis, a KM plotter was applied, with all default settings, and recurrence-free survival (RFS) was preferred, with the auto-best cutoff values and J best probe set. All possible cutoff values between the lower and upper quartiles were determined, and the best presenting threshold was subsequently used as the cutoff. A log-rank *p*-value of <0.05 was considered statistically significant.

## 3. Results

### 3.1. Analysis of Expression Profiles of LSM Family Members

Advances in storing big data, especially banking transcriptomic data, have been surprisingly robust in recent years. Since there are relatively few reports on links between LSM family genes and BRCA, we first identified expression levels of LSMs in BRCA patients using Oncomine. The LSM1 mRNA expression was upregulated in BRCA patients. Similarly, eight databases indicated that LSM4 was overexpressed in BRCA patients. Characteristics of these datasets are displayed in Supplementary Table S1. To derive benefits from clinical data in TCGA, we engaged the DiffExp module of the TIMER server to explore LSM family gene expressions in several cancer types and healthy controls across TCGA datasets. We observed that expressions of LSM1, LSM2, LSM3, LSM4, LSM7, LSM10, LSM12, LSM14A, and LSM14B were higher in tumor samples compared to normal samples. For instance, cohorts of adenoid cystic carcinoma, esophageal carcinoma, BRCA, colon cancer, Lynch syndrome, and lung adenocarcinomas had the most significantly (*p* < 0.001) elevated expressions of LSM1, LSM2, LSM3, LSM4, and LSM5; while LSM7, LSM10, LSM11, LSM12, and LSM14A were significantly overexpressed in stomach adenocarcinoma and uterine corpus endometrial carcinomas compared to healthy samples (Supplementary Figure S1).

**Figure 1:**
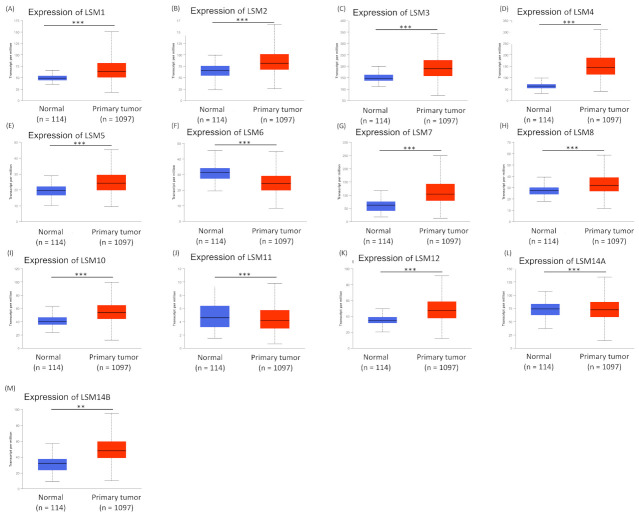
LSMs expressions in patients with breast cancer versus healthy controls (UALCAN database, with 114 normal samples and 1097 primary tumor samples). *p*-value < 0.05 was considered statistically significant by applying Student’s *t*-test. (**A**–**M**) represented the comparison of gene expression between the normal sample and primary tumor of LSM1~LSM14B, respectively. ** *p* < 0.01; *** *p* < 0.001.

We then determined the expression levels of all 13 members of the LSM family in the UALCAN database (Figure 1). The mRNA levels of LSM1, LSM2, LSM3, LSM4, LSM5, LSM7, LSM8, LSM10, and LSM12 were significantly overexpressed in BRCA tissues. In contrast, compared to healthy controls, the transcript levels of LSM6, LSM11 and LSM14A were down-regulated. All *p*-values were <0.01.

### 3.2. Relationships between LSM Family Members and Stages of BRCA

After defining the mRNA expression levels of each LSM member in BRCA associations between their transcriptomic levels of and patients’ tumor stages were analyzed using the UALCAN database. We found significant correlations of LSM1, LSM2, LSM3, LSM4, LSM5, LSM7, LSM10, LSM14B with an uptrend of tumor stages in BRCA patients (Figure S2 in Supplementary). Relationships between the remaining genes of the LSM family and tumor stages were less clear, and statistically non-significant. *p* values are shown in Supplementary Table S2.

### 3.3. Prognosis Value of DNA Methylation of LSM Family Gene

It is widely known as DNA methylation plays an important role in cancer development. Elevated expressions of DNA methyltransferases have been shown in numerous cancers to contribute to tumor growth by methylation-mediated knockdown. [55,56]. We also performed DNA methylation levels in both normal samples (*n* = 97) and primary tumor samples (*n* = 793) of each LSMs family gene in TCGA cohort using UALCAN database (Figure S3 in Supplementary). Methylation levels of LSM2, LSM4, and LSM10 were upregulated in primary breast tumors, while LSM6 methylation levels were downregulated. The remaining LSM family genes were not statistically significant, with a *p*-value of less than 0.05.

### 3.4. LSM Gene Mutations and Co-Expression Analysis

**Figure 2:**
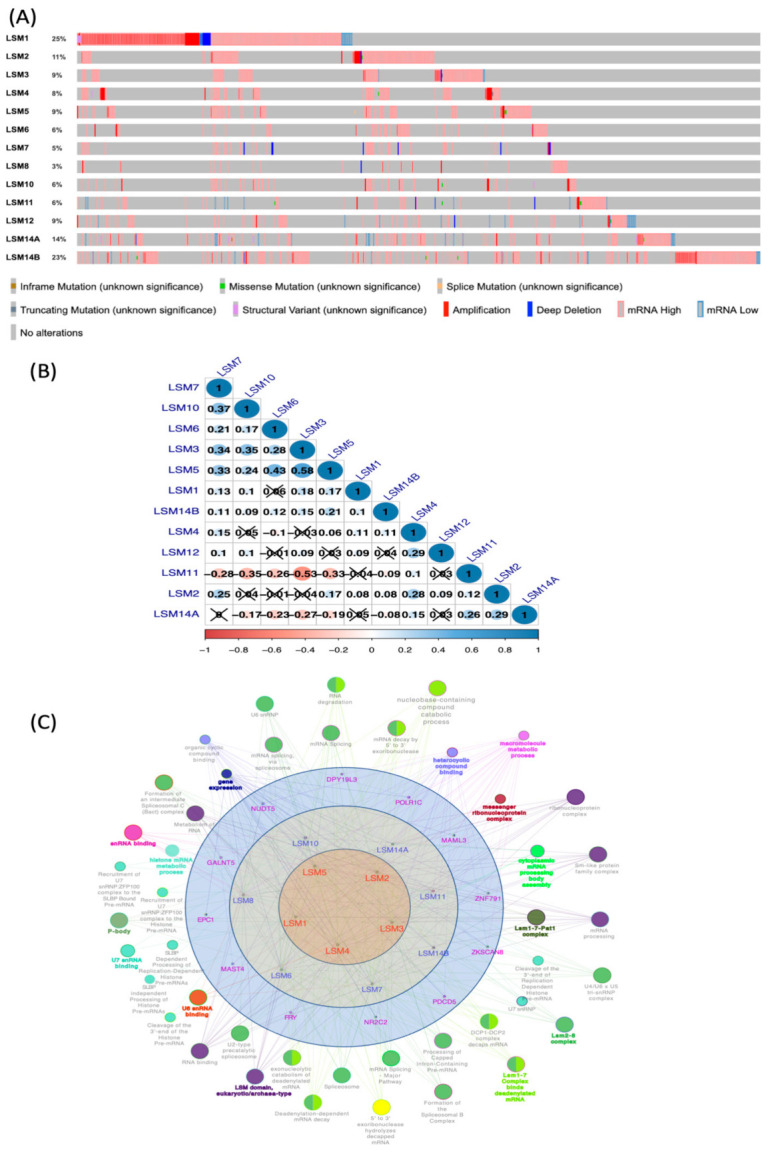
Genetic alterations in LSMs (cBioportal) and co-expression analysis (Cytoscape). (**A**) Summary of alterations in LSMs in TCGA PanCancer Atlas (*n* = 1082). The red bars show gene amplification, blue bars stand for deep deletions, green bars are missense mutations. This plot shows a high alteration rates of LSM1 (25%), LSM2 (11%) LSM4 (8%), LSM14B (23%) (**B**) Correlations among LSM family in breast cancer using TCGA PanCancer Atlas dataset (*n* = 1082). The symmetric correlation matrix was created using the “corrplot” R package. The colors represent the degree of pairwise correlation regarding Spearman’s rank correlation coefficient (rho). Darker blue color and larger dot size mean stronger positive correlation, while darker red indicates higher negative correlation. The Cross symbols represent non-significant correlation coefficient values (*p*-value > 0.01). (**C**) A network of related genes/pathways was constructed. The top 25% of co-expressing genes from the METABRIC database (5000 genes) were collected for each LSM and then intersected with a list of 25 shared genes, and finally input into ClueGo in Cytosape. Only pathways with *p* < 0.05 are shown, with the statistical option as a two-sided hypergeometric test for enrichment and Bonferroni for *p*-value correction.

We investigated mutations of LSM genes in BRCA patients from TCGA Pan-Cancer Atlas (*n* = 1082 patients) in the cBioPortal platform, and found surprisingly high rates of alterations in LSM1 (25%), LSM2 (11%) LSM4 (8%), and LSM14B (23%). We chose the top 25% (5000 genes) from a list of co-expressed genes retrieved from the METABRIC dataset, and then intersected these lists with each gene in the LSM family. Gene ontology (GO) consists of CCs, BPs, and MFs. Results were then input into Cytoscape to build a network of GOs and Kyoto Encyclopedia of Genes and Genomes (KEGG), which are described in detail in Figure 2C. We also evaluated correlations with LSMs by exploring their mRNA expressions, and reported Pearson’s correlation coefficients (Figure 2B).

### 3.5. Survival Analysis of LSM Family Genes

**Figure 3:**
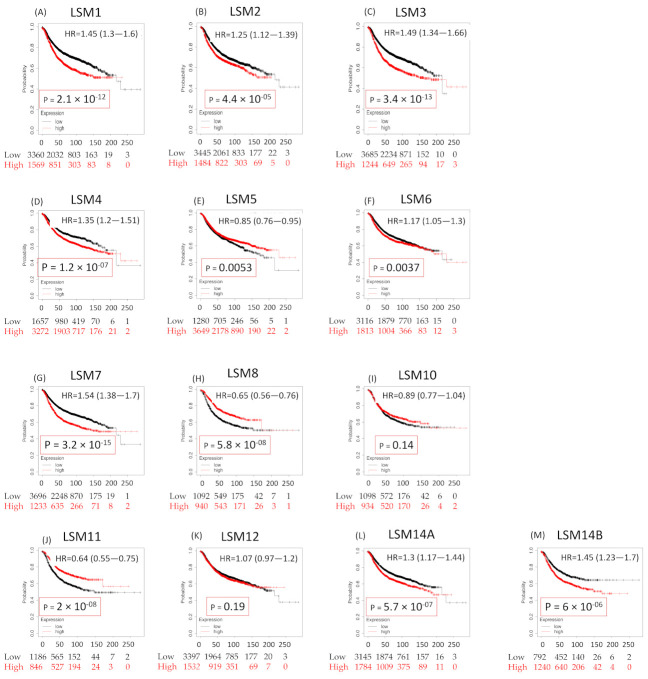
Prognostic value of LSM family genes in breast cancer patients. A recurrence-free survival (RFS) dataset was used for analysis (*n* = 4929 patients). An auto-cutoff strategy was set in this analysis to differentiate patients into two groups based on the value of LSM mRNAs. The best JetSet probes, which describe LSMs, were used to map Affymetrix probe sets by choosing the best probe set for this analysis. Higher expression levels are shown in red, whereas lower expression values are in black. Results showed correlations between expressions of LSMs and survival outcomes of breast cancer patients. By splitting patients by the median, only the best probe Jetset and auto-cutoff were queried. The results indicated that LSM1/2/3/4/5/6/7/14A/14B were significantly associated with poor prognostic outcome of breast cancer patients.

We evaluated relationships between LSM mRNA expression levels and survival rates of BRCA patients by performing a KM analysis to uncover the prognostic value of each LSM. The results indicated that 8 of 13 members of the LSM family were significantly associated with poor prognostic outcomes of BRCA patients with respect to RFS, such as LSM1 (HR = 1.45, 95% CI: 1.3–1.6, *p* for trend = 2.1 × 10^−12^), LSM2 (HR = 1.25, 95% CI: 1.12–1.39, *p* for trend = 4 × 10^−5^), LSM3 (HR = 1.49, 95% CI: 1.34–1.66, *p* for trend = 3.4 × 10^−13^), LSM4 (HR = 1.35, 95% CI: 1.21–1.51, *p* for trend = 3.4 × 10^−7^), LSM6 (HR = 1.17, 95% CI: 1.05–1.29, *p* for trend = 0.0037), LSM7 (HR = 1.54, 95% CI: 1.38–1.72, *p* for trend = 3.2 × 10^−15^), LSM14A (HR = 1.3, 95% CI: 1.17–1.44, *p* for trend = 5.7 × 10^−7^), and LSM14B (HR = 1.45, 95% CI: 1.23–1.7, *p* for trend = 6 × 10^−6^). In contrast, low expression levels of LSM5, LSM8, and LSM11 indicated longer recurrence metastasis-free survival (HR = 0.85, 95% CI: 0.76–0.95, *p* for trend = 0.0053, HR = 0.85, 95% CI: 0.56–0.76, *p* for trend = 5.6 × 10^−8^, HR = 0.64, 95% CI: 0.56–0.75, *p* for trend = 2 × 10^−8^, respectively). In addition, LSM10 and LSM12 showed non-significant prognostic values. The results are tabulated Figure 3. Subsequent to the differential expression analysis and survival analysis, we decided to select LSM1, LSM2, LSM3, LSM4, LSM7, and LSM14B for further exploration, due to the following reasons. First, these genes showed high expression levels in breast tumors compared to normal samples. Second, when spotting the stages of cancer development, significantly increased mRNA values in higher-level tumor stages should be detected. Third, reflecting the prognostic values, higher expression levels of these family genes should lead to poorer survival outcomes. Collectively, after comparing results from the survival analysis and relationships between expression levels of LSM family genes and normal tissues in terms of individual stages, we observed that LSM1, LSM2, LSM3, LSM4, LSM7, and LSM14B all fit the above criteria.

### 3.6. Protein Expressions of LSM Family Members

**Figure 4:**
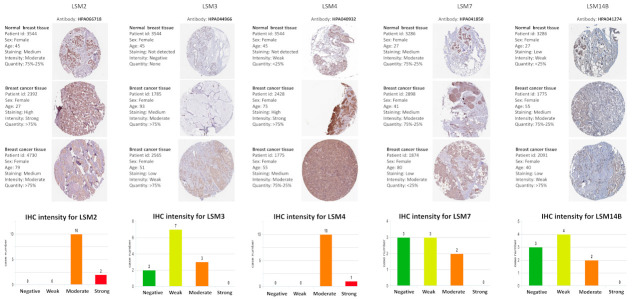
Representative IHC images of LSM2, LSM3, LSM4, LSM7, and LSM14b in breast cancer patients, which showed normal and tumor samples (Human Protein Atlas) and IHC intensities of these genes. Bar charts represent IHC staining intensities of LSM2 (12 patients), LSM3 (12 patients), LSM4 (11 patients), LSM7 (8 patients), and LSM14B (9 patients). All IHC images and patient information were obtained from the Human Protein Atlas.

We investigated the protein expression of LSMs in the Human Protein Atlas database (Figure 4). The immunohistochemstry images of LSM2, LSM3, LSM4, LSM7, LSM14b in breast cancer patients, including their clinicopathological parameters such as Patient ID, Gender and Age, which showed the normal and tumor samples (Human Protein Atlas) were presented in Figure S4 in Supplementary. In this analysis, results of LSM1 protein expression were not found due to the absence of antibodies. LSM2 and LSM4 proteins were overexpressed in tumor tissues with similar patterns in BRCA patient samples in the HPA dataset. Meanwhile, we found that LSM7 and LSM14B proteins were not significantly differentially expressed between normal and cancer tissues. Of note, these results showed the same trend as the mRNA expression profiles. Protein expression score is determined by manually scoring immunohistochemical data for staining intensity (negative, weak, moderate, or strong) and proportion of stained cells (25 percent, 25–75 percent, or >75 percent). The following formula is used to automatically translate each intensity and fraction combination into a protein expression level score: negative-not detected; weak <25%–not detected; weak combined with either 25–75% or 75%–low; moderate <25%–low; moderate combined with either 25–75% or 75%–medium; strong <25%–medium, strong combined with either 25–75% or 75%–high.

### 3.7. Relationship between LSMs Transcriptomic Expression Levels and Biomarkers of Different Immune Cells

**Figure 5:**
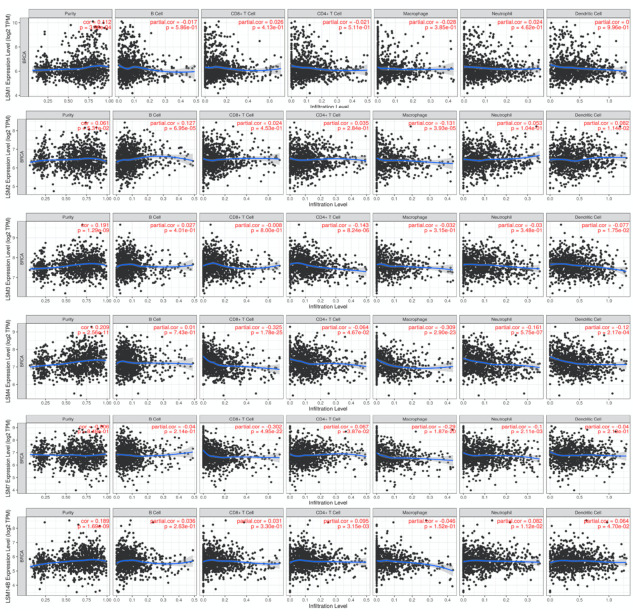
Correlations of LSM1, LSM2, LSM3, LSM4, LSM7, and LSM14B expressions with immune infiltration profiles in breast cancer (from TCGA database). The figures show the each LSM gene expression was associated with tumor purity and several tumor-infiltrating immune cell markers, namely CD8+ T cells, B cells, CD4+ T cells, neutrophils, macrophages, and dendritic cells. Spearman correlations were applied to describe correlations between LSM family genes and the above-mentioned immune cells (*p* < 0.05 was accepted as statistically significant).

**Figure 6:**
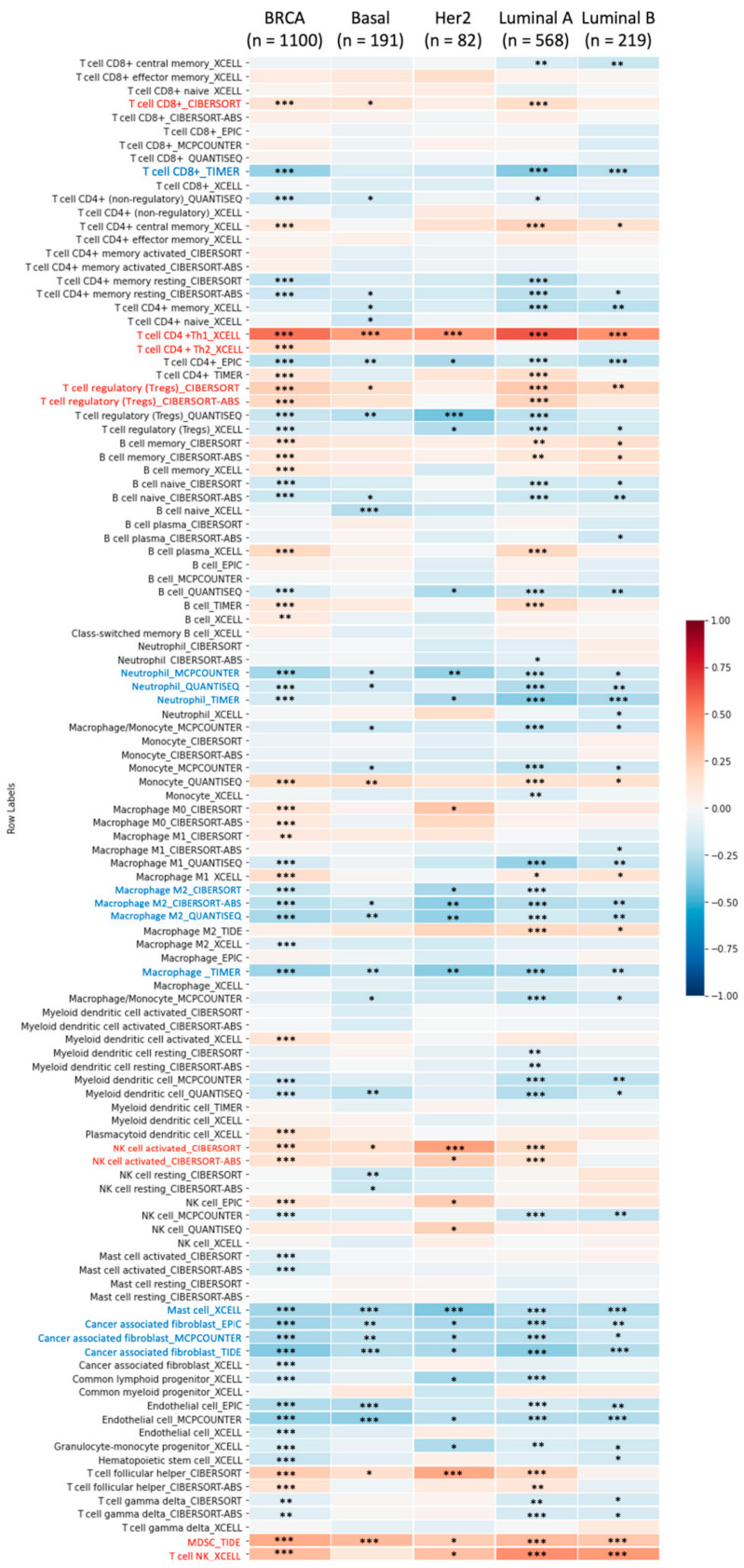
Heatmap of LSM4 expression and immune infiltrates in various breast cancer subtypes. The plot indicates correlations of breast cancer subclasses (basal, HER2, luminal A, and luminal B) and the number of samples out of 112 immune infiltrates methods from six state-of-the-art algorithms, consisting of TIMER, EPIC, CIBERSORT, xCell, MCP-counter, and quanTIseq. R-scores ranged −1.0–1.0. A value of *r* = 1 denotes a perfect positive correlation, while a value of *r* = −1 shows a perfect negative correlation. (* *p* < 0.05, ** *p* < 0.01, *** *p* < 0.001).

An evolving TME is a convoluted and continually growing entity. The configuration of the TME varies among tumor types. A large body of research has revealed the important role of the TME in cancer progression [57,58,59,60]. We further evaluated correlations between LSM expressions and biomarkers of tumor-infiltrating immune cells, namely B cells, M1 macrophages, tumor-associated macrophages (TAMs), neutrophils, M2 macrophages, and dendritic cells (DCs). LSM1 expression showed a significant positive correlation with tumor purity (*r* = 0.112, *p* = 3.96 × 10^−4^). LSM2 was significantly associated with B cells (*r* = 0.127, *p* = 6.95 × 10^−5^) and macrophages (*r* = −0.131, *p* = 3.93 × 10^−5^). Similarly, LSM3 expression was correlated with tumor purity (*r* = 0.191, *p* = 1.29 × 10^−9^) and CD4^+^ T-cell markers (*r* = −0.143, *p* = 8.24 × 10^−6^). LSM4 expression exhibited positive correlations with tumor purity (*r* = 0.209, *p* = 2.56 × 10^−11^), CD8^+^ T cells (*r* = −0.325, *p* = 1.78 × 10^−25^), macrophages (*r* = −0.309, *p* = 2.9 × 10^−23^), neutrophils (*r* = −0.161, *p* = 5.75 × 10^−7^), and DCs (*r* = −0.12, *p* = 2.17 × 10^−4^). LSM7 was associated with CD8^+^ T-cell markers (*r* = −0.302, *p* = 4.95 × 10^−22^), macrophages (*r* = −0.29, *p* = 1.87 × 10^−20^), and neutrophil markers (*r* = −0.1, *p* = 2.11 × 10^−3^). LSM14B was correlated with purity (*r* = 0.189, *p* = 1.69 × 10^−9^) and CD4^+^ T-cell markers (*r* = 0.095, *p* = 3.15 × 10^−3^) (Figure 5) Eventually, through an HPA analysis, we observed a significant association between LSM4 expression in breast tumors and staining samples, while those of the remaining genes remained less clear, hence suggesting that aside from LSM4, those genes would have limited roles as prognostic biomarkers for BRCA. Furthermore, when exploring correlations between immune cells and cancer cells in the TME, we discovered that not only was LSM4 expressed by cancer cells, but most immune cells in several immune deconvolution methods also infiltrated BRCA tumors and subtypes with high expression of LSM4. In particular, results for CD4^+^ T cells revealed that LSM4 had strong positive correlations in TIMER (*r* = 0.129, *p* = 4.83 × 10^−5^) and XCELL (*r* = 0.122, *p* = 1.14 × 10^−4^), while CD4^+^ Th1 T cells were highly correlated with basal (*n* = 191, *r* = 0.415, *p* = 1.25 × 10^−08^), human epidermal growth factor receptor 2 (HER2) (*n* = 82, *r* = 0.438, *p* = 1.22 × 10^−4^), luminal A (*n* = 668, *r* = 0.621, *p* = 2.06 × 10^−56^), and luminal B subtypes (*n* = 219, *r* = 0.454, *p* = 3.95 × 10^−11^). On the other hand, LSM4 was positively correlated with CD8^+^ T cells in CIBERSORT methods in most BRCA subtypes (BRCA: *r* = 0.151, *p* = 1.67 × 10^−6^; basal: *r* = 0.159, *p* = 3.67 × 10^−2^; and luminal A: *r* = 0.185, *p* = 32.37 × 10^−5^); as well as T cell follicular helpers in CIBERSORT (BRCA: *r* = 0.254, *p* = 3.89 × 10^−16^; basal: *r* = 0.164, *p* = 3.04 × 10^−2^, HER2: *r* = 0.388, *p* = 7.51 × 10^−4^, and luminal A: *r* = 0.233, *p* = 8.32 × 10^−8^). In contrast, we found that it had negative correlations with M2 macrophages in CIBERSORT-ABS (BRCA: *r* = −0.229, *p* = 2.78 × 10^−13^; basal: *r* = −0.188, *p* = 1.29 × 10^−2^; HER2: *r* = −0.348, *p* = 2.76 × 10^−3^; luminal A: *r* = −0.256, *p* = 3.2 × 10^−9^; and luminal B: *r* = −0.232, *p* = 1.23 × 10^−3^) and neutrophils in MCP-COUNTER (BRCA: *r* = −0.294, *p* = 3.16 × 10^−21^; basal: *r* = −0.195, *p* = 1.00 × 10^−2^; HER2: *r* = −0.325, *p* = 5.36 × 10^−3^; luminal A: *r* = −0.214, *p* = 8.72 × 10^−7^; and luminal B: *r* = −0.174, *p* = 1.6 × 10^−2^). Results are described in Figure 6. Therefore, we further explored LSM4 by investigating its biological functions and activities of gene products and complexes through a GO analysis.

### 3.8. Comprehensive Results of LSM4 in Functional Enrichment Analysis

#### 3.8.1. Gene Ontology Enrichment Analysis

**Figure 7:**
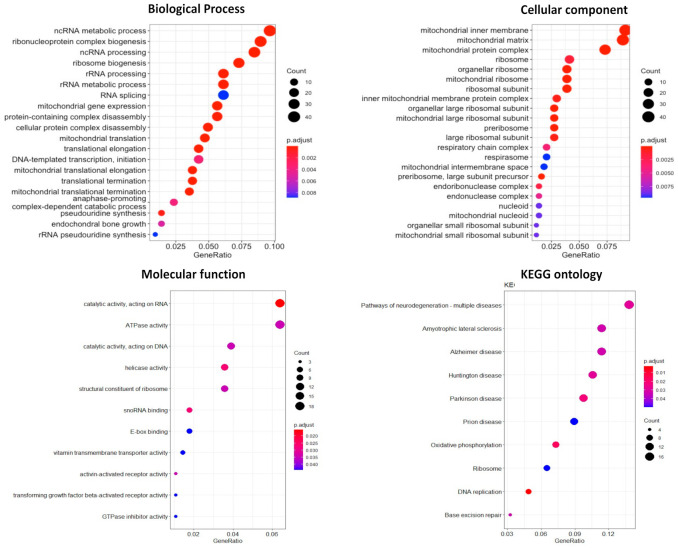
Gene ontology (GO) terms (biological processes, cellular components, molecular functions, and KEGG) with *p* value of genes. Circle sizes represent the number of genes counted in each function, and bubble colors correspond to *p* values.

For comprehensive exploration, we extracted data from METABRIC and TCGA Pan-Cancer Data to retrieve GO enrichment results, including CCs, BPs, MFs, and KEGG. For BPs, we observed that LSM4 was correlated with non-coding (nc)RNA metabolic processes and ribonucleoprotein complex biogenesis. In contrast, the CC analysis showed localization in mitochondria, such as the mitochondrial inner membrane, mitochondrial matrix, and mitochondrial complex. Finally, from MF results, “catalytic activity, acting on RNA” and “ATPase activity” were strongly associated with high LSM4 expression in BR tumors, while KEGG ontology indicated the role of pathways of neurodegeneration-multiple diseases, as well as other disease-related pathways, such as amyotrophic lateral sclerosis, Alzheimer’s disease, Huntington disease, and Parkinson’s disease (Figure 7).

#### 3.8.2. High Expression of LSM4 Is Related to Epithelial-Mesenchymal Transition (EMT) and Pro-Cancerous Related Gene Sets in Breast Cancer

**Figure 8:**
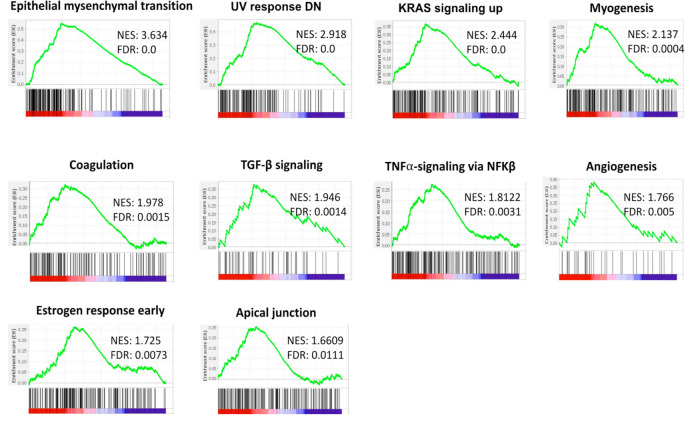
Results of gene set enrichment assay (GSEA) in high expression of LSM4 in breast cancer patients in TCGA cohort. From TCGA Pan-Cancer dataset, patients were split into two groups with low or high LSM4 mRNA expression; then a related ranked genes list was obtained and input to GSEA. From the GSEA software, statistical significance was considered as an FDR value < 0.25, normalized enrichment score (NES) > 1.5, and nominal *p*-value < 0.05, which was recommended by GSEA database. A positive NES value, which reflects the enrichment pathway in the list, represents the enrichment at the top of pathways.

It was interesting to discover underlying biological processes of gene sets which were co-expressed with LSM4. A GSEA was utilized to investigate the enrichment of MSigDB Hallmark gene sets in BRCA samples with high expression levels of LSM4. We found that the EMT, coagulation, tumor necrosis factor (TNF)-α-signaling via nuclear factor (NF)-κβ, interleukin (IL)-2/signal transduction and activator of transcription 5 (STAT5) signaling, apical junction, and androgen response, which are known as inflammation- and immune-related gene sets, were enriched with high LSM4 expression in breast tumors. Moreover, LSM4 was also significantly expressed in a procancerous gene set, such as KRAS signaling upregulation, angiogenesis, and transforming growth factor (TGF)-β signaling (Figure 8). The detailed enrichment results are shown in Supplementary Data.

#### 3.8.3. Identification of Differentially Expressed Genes (DEGs) in BRCA Patients

It was interesting to validate our results in independent datasets to evaluate the consistency of our results. In particular, using the GDC dataset, comparisons of LSM4 expression levels in normal tissues and tumor tissues were described via a violin plot, with a *t*-test *p*-value of <2.2 × 10^−16^. We observed a congruous outcome with our results above, revealing the significantly high expression of LSM4 in BRCA patients (Supplementary Figure S5). Moreover, a heatmap was conducted to clearly describe the relationship of LSM4 expression levels between two different phenotypes: normal and tumor tissues. The analysis was based on log2[fold of change] values, showing a list of 60 genes over the entire genome, and its expression profiles. We found that LSM4 was also overexpressed in tumor samples, and underexpressed in normal controls (Figure S6 in Supplementary). Interestingly, we observed that LSM4 was closely ranked with other breast cancer biomarkers, such as DPP3 [51], CDK5 [61], and TRIB3 [62]. These biomarkers showed consistent results of high expression levels in tumors and low expression levels in normal phenotypes.

Additionally, it is widely known that DNA methylation plays a crucial role in cancer development. In many malignancies, elevated expressions of DNA methyltransferases have been shown to contribute to tumor growth by methylation-mediated gene inactivation. We performed a heatmap of the various DNA methylated locations of LSM4, and DNA methylation-based survival studies using the TCGA dataset (Figure S7 in Supplementary). A total of 18 methylated CpG sites of LSM4 were found using the MethSurv database, with nearly half of the CpG sites having predictive relevance in breast cancer patients. Among them, cg26961332 showed the highest level of DNA methylation. This result provides a potential mechanism by which LSM4 serves as an oncogene for breast cancer. The comprehensive values of CpGs in LSM4 are shown in Tables S3 and S4, Supplementary.

Accumulation of changes in tumor-suppressor genes and oncogenes was highly correlated with the occurrence and development of tumors [63]. In this study, we utilized a DEG method using R packages to validate our results of the roles of LSM4 expression in GO and KEGG pathway analyses. Consistently, in terms of BPs, the results showed that high levels of DEGs were highly enriched in ncRNA metabolic processes, ribonucleoprotein complex biogenesis, and ncRNA processing. Similarly, regarding MFs, upregulated DEGs were involved in catalytic activity by acting on RNA; while in terms of CCs, the mitochondrial inner membrane and mitochondrial matrix were highly related to upregulated DEGs. In addition, the KEGG analysis illustrated the role of overexpressed LSM4 in the pathway of neurodegeneration-multiple disease, Alzheimer’s disease, etc. Results are shown in Supplementary Materials, Figure S8.

#### 3.8.4. LSM4 Not Only Plays Roles in BRCA Development but Is Also Involved in Various Cancer Types

**Figure 9:**
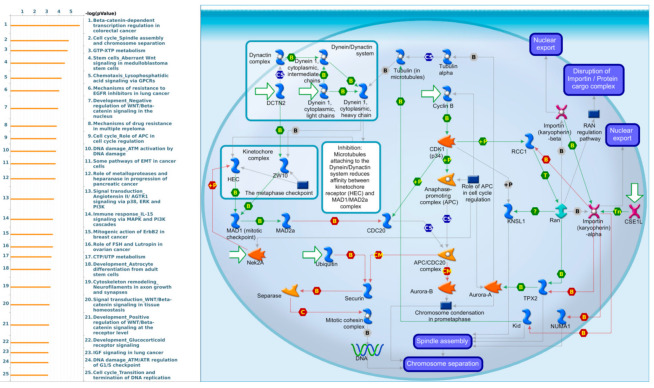
MetaCore enrichment pathway analysis of LSM4 co-expressed gene list in a breast cancer database. The approach was to collect the top 10% of expression gene lists from both METABRIC (2000 genes) and Pan-Cancer (1800 genes), then overlap them to produce a final genes list of 463 total genes. The pathway list was ordered by the -log [*p* value] from the gene list extracted via TCGA Pan-Cancer atlas and METABRIC breast cancer patient databases. “Cell cycle Spindle assembly and chromosome separation” was at the top of the pathway list when performing the biological process analysis.

MetaCore is commonly employed to construct pathways networked from an input gene list to stimulate BPs. After setting the gene list from the intersection of TCGA and METABRIC datasets as input for the MetaCore analysis, we identified interesting results related to LSM4. In particular, it was closely related to many types of cancer signaling pathways, such as “Beta catenin-dependent transcription regulation in colorectal cancer”, “Stem cells aberrant Wnt signaling in medulloblastoma stem cells”, “Mechanism of resistance to EGFR inhibitors in lung cancer”, and “Mechanism of drug resistance in multiple myeloma”. Meanwhile, LSM4 was also correlated with “Immune response IL-15 signaling via MAPK and PI3K cascade”, “Cell cycle spindle assembly and chromosome separation”, and “Mitogenic action of ErbB2 in breast cancer”, which are immune- and cell cycle-related pathways that play roles in BRCA growth. The pathway list and networks are shown in Figure 9 and Table S5, Supplementary.

## 4. Discussion

The incidence of BRCA has been significantly increasing every year, making it one of the most common cancers in women worldwide [64,65,66]. For decades, although there have been astonishing efforts to improve the efficacy of BRCA treatments and prognoses, its biology has not yet been fully elucidated. Currently, most patients are diagnosed based on widespread mammogram screening programs. However, in nearly one-third of patients, cancerous growth has already spread to regional lymph nodes at the time of diagnosis [67]. Therefore, it is necessary to investigate novel prognostic techniques for early-stage detection, which employ new biomarkers in a vital role [68,69,70,71,72,73]. Furthermore, characterizing the immune system, profiling the TME, and constructing BRCA immunotherapies are also known as critical keys in cancer research [74,75].

In this study, several analyses of LSM family genes were conducted. LSMs were previously known as U6 small nuclear RNA and mRNA degradation-associated protein-coding genes, and are expressed in multiple cell lines and human organs [76,77,78]. Our goal was to thoroughly analyze the BPs of LSMs in BRCA by performing a comprehensive analysis based on public databases. We commenced by using high-throughput techniques to analyze the role of LSM family genes, by comparing normal to cancer cells, and their relevance to signaling pathways in BRCA development. We were able to identify interesting findings for each individual gene, and further evaluated targeted therapeutic approaches. To our knowledge, this is the first study to apply multiple bioinformatics strategies to explore associations between expressions of *LSM* family genes and comparative clinicopathological parameters in BRCA patients. We suggest that LSMs could serve as novel biomarkers in BRCA.

Oncogenes are the key genes that contribute to the transformation of normal cells into malignant cells, whereas tumor-suppressive genes prevent the development of the cancer. Tumor formation and progression are defined by individual processes that collaborate, and a greater knowledge of each individual process may give a better foundation for future anticancer research [79]. The emergence of large available datasets around the world have required the use of robust high-throughput analysis to interpret them. In this study, we conducted genes expression of LSMs family in over 20 types of cancer by screening the gene names and applying the thresholds on Oncomine. LSM1, LSM2, LSM3, LSM4, LSM5, LSM7, LSM12, and LSM14B were overexpressed in breast cancer samples compared to normal tissues, LSM6 and LSM11 were underexpressed, whereas LSM8, LSM14A did not show significant upregulations. It was previously determined that LSM1, an oncogene when working in combination with BAG4 and C8orf4, can influence growth factors and affect phenotypes in human mammary epithelial cells [80,81]. Additionally, higher expression of LSM1 resulted in a higher abundance of hepatic metastatic lesions, due to previous report [82]. Consistently, LSM1 has been found as an oncogene activated by gene amplification and it could play a crucial role in breast cancer development and progression [23]. Pan et al. has studied the role of LSM2 in lung cancer development [83]. LSM3 was found to be downregulated in cervical cancer and associated with poor progression-free survival outcome [84,85]. Those reports showed a reverse compare to our survival analysis in breast cancer patients. This inconsistency could be explained by the variability of datasets and the need for further in vitro or in vivo investigations. In colorectal cancer, LSM3 was also significantly associated with lymphatic metastasis [86], however, similar reports in breast cancer are lacking. LSM4 has known as a member of the LSM family of RNA-binding proteins, plays an important role in pre-mRNA splicing by mediating U4/U6 snRNP formation, and involved in pancreatic cancer [87,88]. Indeed, Long et al. demonstrated the effect of RNA-binding protein LSM4 on the growth and locomotion of esophageal cancer cells [89]. A study of Ho et al. showed the correlation between expression of LSM4 and ovarian cancer [90]. Interestingly, Yin et al. reported that LSM4 significantly overexpressed in triple-negative breast cancer (TNBC) patients compared to other breast cancer subtypes, which strongly support our conclusion in this study [91]. In contrast, Wang et al. found a consistent trend of LSM6 expression, which was down-regulated in Basal-like breast cancer subtype [92]. For the other genes in LSM family, there are lack of evidence of their roles in cancer phenotypes. From our point of view, the different trends of LSMs expression in specific cancer types could be explained by genetic compensation [93,94]. In particular, protein post-translational modifications (PTM) are reported to crosstalk among each other, resulting in complex phenotypic outcomes. Of interest, our findings through DNA methylation level analysis by using UALCAN database also support the consistent trend, which show LSM2, LSM4, LSM10 were upregulated in primary breast tumors, while LSM6 methylation levels were downregulated.

For more in-depth analysis, we utilized the expression profile of each gene in order to find correlations with different stages of BRCA. We found that LSM1 expression was significantly correlated with tumor stages and related to a worse distant-metastasis survival rate. Interestingly, its mutation rate was 25%, suggesting that this frequency of new mutations in BRCA is high, and might not be rate-limiting for developing tumors [95]. It was found that LSM1 was associated with the “cytoskeleton remodeling regulation of actin cytoskeleton organization by the kinase effectors of Rho GTPases” pathway. Several studies spanning decades revealed that Rho GTPases play essential roles in assorted cellular events such as cell growth control, membrane trafficking, and transcriptional regulation [96]. Moreover, LSM1 was highly correlated with “Chemoresistance pathways mediated by constitutive activation of PI3K pathway and BCL-2 in small cell lung cancer” and “IGF-1 receptor/EGFR cooperation in lung cancer”, which suggested that it also plays an important role in lung cancer tumor growth.

In this study, we identified a significant correlation between the overexpression of LSM2 and increasing tumor stages. Furthermore, when extracting the co-expression gene list from TCGA and METABRIC datasets, we observed that LSM2 was closely co-expressed with various breast cancer biomarkers such as PDCD5 [97] and NUDT5 [98]. In a survival analysis, it was also indicated that LSM2 was related to a poor RFS prognosis. As a member of the LSM family, the role of LSM2 in “cell cycle--role of APC in cell cycle regulation” was established in this study. The anaphase-promoting complex (APC) is known as a ubiquitin ligase, generally required to give rise to progression and exit from mitosis by producing proteolysis of diverse cell cycle regulators. VanGenderen et al. described the key role of the APC in BRCA tumor development and progression [99], reporting that finding an inhibitor of cancer growth activities is a useful approach to treating cancer. Consistently, another report showed that LSM2, a RNA splicing gene, was upregulated in basal-like primary tumors, including BRCA [100].

When conducting a survival analysis, we observed that high LSM3 expression levels were significantly correlated with shorter survival times in patients with BRCA. Of interest, via a TIMER analysis, LSM3 showed a strong relationship with CD4^+^ T cells, which facilitate anticancer immunity mostly by offering help for CD8^+^ T cell and antibody responses, and via releasing effector cytokines including interferon (IFN)-γ and tumor necrosis factor (TNF)-α, which so far are known to play vital roles in antitumor immunity [101]. In addition, MetaCore pathway maps revealed that LSM3 was mainly correlated with ubiquinone metabolism. A previous study by Chan et al. demonstrated a correlation between ubiquinone and metabolic disorders in patients with oral cancer [102]. For the first time, our study showed that ubiquinone metabolism could have a potential role in BRCA development.

Similarly, LSM7 was also found to be upregulated in BRCA patients at different stages. Moreover, the KM plot results showed that BRCA patients with high LSM7 expression had a significantly poorer survival rate compared to a control group. A previous study revealed that triple-negative breast cancer cells react to T cells at the splicing layer, where LSM7 is located [103]. This finding is consistent with our study, which showed that LSM7 had significant relevance to CD8^+^ T-cell markers, macrophages, and neutrophil markers in a TME analysis. In cancer, neutrophils play major roles in inflammatory functions and in innate and adaptive immunity, and are dynamically involved in progression and metastasis, hence serving as emerging targets for multiple cancer types [104,105]. Furthermore, LSM7 was associated with “regulation of actin cytoskeleton nucleation and polymerization by Rho GTPase”, a pathway that plays an important role in the movement of cancer cells in living tumor tissues [106]. In vivo, groups of cytoskeletal proteins are often overexpressed in cancer cells, while in vitro they play roles in cancer cell migration and invasion, as reported in a recent study [107].

There have been no studies determining the precise role of LSM14B in any disease. In this report, we observed evidence of LSM14B overexpression that affected outcomes of the survival analysis and increasing expression levels in more progressive tumor stages. LSM14B showed a relationship with the BP of “Inhibition of oligodendrocyte precursor cell differentiation by Wnt signaling in multiple sclerosis”, a pathway involved in chronic demyelinating diseases. Therefore, additional work is required to determine the precise role of LSM14B in BRCA.

Our method provides an observation of correlations between LSM4 expression and BRCA tumor, and revealed that LSM4 overexpression was associated with progressive stages of BRCA. Furthermore, the HPA showed that LSM4 had a moderate to strong IHC intensity in BRCA samples compared to normal breast tissues. From the results of a TME analysis, CD8^+^ T cells, macrophages, neutrophils, and DCs were strongly associated with LSM4 in targeting cancer cells, leading to a potential role of LSM4 in immunotherapy. Consistently, previous studies demonstrated that macrophages are widely present in solid tumors [108,109] and are a fundamental driver of cancer growth and metastasis [110]. However, only a small percentage of individuals have had a positive clinical response to such therapies. Understanding the cellular proportions, heterogeneity, and geographic distributions of the tumor immune milieu is thought to aid in better stratifying patients who would benefit from immunotherapeutics [111]. While BRCA was previously regarded as relatively non-immunogenic, it is now suggested that BRCA is in fact rich in immune infiltrates, with various functions and prognostic values [112]. Tumor-infiltrating lymphocytes (TILs) are chiefly represented by T cells (CD3^+^) and consist of CD4^+^, CD8^+^. and T-regulatory (Treg) cells [113]. DCs, CD4^+^, and CD8^+^ T cells, and a minor component of TILs were represented by B cells and plasma cells. Our results in Figure 8 show that high LSM4 expression was positively correlated with CD4^+^ T cells, including type 1 T-helper (Th1) cells in most BRCA subtypes. Consistent with previous studies, it increases the antitumor activity of NK cells and macrophages [114]. In addition, Treg cells, which are involved in cancer growth by inhibiting anticancer immunity [115], were also highly correlated with LSM4, especially in the luminal A subclass. A previous report also demonstrated that Treg lymphocyte infiltration plays a role in metastatic BRCA [116], consistent with our findings. Furthermore, the HER2 and luminal A subtypes were also significantly and positively correlated with NK cells and cytotoxic lymphocytes, which participate in innate immunity and are capable of detecting and killing tumor cells, similar to findings by Verma et al. [117]. Interestingly, we found that high LSM4 expression was associated with myeloid-derived suppressor cells (MDSCs), which were suggested to constitute a tumor-favoring microenvironment [118,119] in basal, and luminal A and B subtypes. In a previous study, Chen et al. revealed the promoting role of expressing MDSCs in basal-like transition and metastasis of BRCA, which is similar to our report [120]. Among several signaling pathways, LSM4 was significantly correlated with “Beta catenin-dependent transcription regulation in colorectal cancer”, consistent with a previous study [121]. LSM4 was found to be related to diverse cancer-type signaling pathways, such as “Stem cells aberrant Wnt signaling in medulloblastoma stem cells”, “Mechanism of resistance to EGFR inhibitors in lung cancer”, and “Mechanism of drug resistance in multiple myelomas”, suggesting its important role in cancer development. Therefore, LSM4 is not only associated with BRCA, but is also involved in various other types of cancer.

## 5. Conclusions

In summary, while many observations have demonstrated that LSM2, LSM3, LSM7, and LSM14B play decisive roles in BRCA development, further assessments to verify these findings in BRCA tumors are warranted. Using meta-analysis and combbine with bioinformatics approach, our study suggested that among LSM family genes, LSM4 has prospective value and may serve as a new prognosticator and therapeutic target for BRCA treatment. The main drawback of the present study is the retrospective nature of transcriptomic analyis, which requires further confirmation in a larger prospective study to confirm whether LSM4 expression can be recognized as a useful biomarker in clinical practice.

## Data Availability

CBioportal: https://cbioportal.org (accessed on 1 May 2021).; The Human Protein Atlas: https://www.proteinatlas.org (accessed on 1 May 2021); Kaplan Meier-plot database https://kmplot.com (accessed on 1 May 2021), Metacore Analysis https://portal.genego.com (accessed on 1 May 2021). The datasets used and/or analyzed during the current study are available from the corresponding author on reasonable request.

## Supplementary Materials

The following are available online at https://www.mdpi.com/article/10.3390/cancers13194902/s1, Figure S1: LSM family genes expression in several cancer types, Figure S2: LSMs transcription levels in different stages of breast cancer (UALCAN database), Figure S3: LSMs DNA methylation level in patients with breast cancer versus healthy controls, Figure S4: Representative immunohistochemistry images of LSM2, LSM3, LSM4, LSM7, LSM14b in breast cancer patients, Figure S5: LSM4 expression between normal and tumor groups (*n* = 1222 patients from GDC database), Figure S6: Expression profiles of LSM4 in DEGs methods, Figure S7: DNA methylation clustered expression of LSM4 (TCGA dataset), Figure S8: Functional enrichment analysis with gene ontology (GO) terms and KEGG of differentially expressed genes (DEGs); Table S1: The basic characteristic of LSM4 gene on Oncomine database, Table S2: Statistical values of LSM family genes expression base on individual cancer stage, Table S3: Prognosis value of CpGs in LSM4 (MethSurv database), Table S4: Gene sets enriched in high expression LSM4, Table S5: Pathway analysis of LSM4-coexpressed genes from public breast cancer databases using the MetaCore database.

## References

[1] (2021). Breast Cancer.

[2] Azubuike S.O., Muirhead C., Hayes L., McNally R. (2018). Rising global burden of breast cancer: The case of sub-Saharan Africa (with emphasis on Nigeria) and implications for regional development: A review. World J. Surg. Oncol..

[3] Ginsburg O., Bray F., Coleman M.P., Vanderpuye V., Eniu A., Kotha S.R., Sarker M., Huong T.T., Allemani C., Dvaladze A. (2017). The global burden of women’s cancers: A grand challenge in global health. Lancet.

[4] Susini T., Saccardin G., Renda I., Giani M., Tartarotti E., Nori J., Vanzi E., Pasqualini E., Bianchi S. (2021). Immunohistochemical evaluation of FGD3 expression: A new strong prognostic factor in invasive breast cancer. Cancers.

[5] Renda I., Bianchi S., Vezzosi V., Nori J., Vanzi E., Tavella K., Susini T. (2019). Expression of FGD3 gene as prognostic factor in young breast cancer patients. Sci. Rep..

[6] Susini T., Berti B., Carriero C., Tavella K., Nori J., Vanzi E., Molino C., Di Tommaso M., Santini M., Saladino V. (2014). Topoisomerase II alpha and TLE3 as predictive markers of response to anthracycline and taxane-containing regimens for neoadjuvant chemotherapy in breast cancer. OncoTargets Ther..

[7] Thorat M.A., Balasubramanian R. (2020). Breast cancer prevention in high-risk women. Best Pract. Res. Clin. Obstet. Gynaecol..

[8] Lin C.Y., Lee C.H., Chuang Y.H., Lee J.Y., Chiu Y.Y., Wu Lee Y.H., Jong Y.J., Hwang J.K., Huang S.H., Chen L.C. (2019). Membrane protein-regulated networks across human cancers. Nat. Commun..

[9] Tsai H.T., Huang C.S., Tu C.C., Liu C.Y., Huang C.J., Ho Y.S., Tu S.H., Tseng L.M., Huang C.C. (2020). Multi-gene signature of microcalcification and risk prediction among Taiwanese breast cancer. Sci. Rep..

[10] Nguyen H.D., Liao Y.C., Ho Y.S., Chen L.C., Chang H.W., Cheng T.C., Liu D., Lee W.R., Shen S.C., Wu C.H. (2019). The α9 Nicotinic acetylcholine receptor mediates nicotine-induced PD-L1 expression and regulates melanoma cell proliferation and migration. Cancers.

[11] Lee K.L., Kuo Y.C., Ho Y.S., Huang Y.H. (2019). Triple-negative breast cancer: Current understanding and future therapeutic breakthrough targeting cancer stemness. Cancers.

[12] Nuffiedtrust (2021). Cancer Survival Rates.

[13] Villasco A., Accomasso F., D’Alonzo M., Agnelli F., Sismondi P., Biglia N. (2021). Evaluation of the ability of the Clinical Treatment Score at 5 years (CTS5) compared to other risk stratification methods to predict the response to an extended endocrine therapy in breast cancer patients. Breast Cancer.

[14] Villasco A., Agnelli F., D’Alonzo M., Accomasso F., Sismondi P., Biglia N. (2021). Validation of CTS5 on a retrospective cohort of real-life pre- and postmenopausal patients diagnosed with estrogen receptor-positive breast cancers: Is it prognostic?. Clin. Breast Cancer.

[15] Modaffari P., Ponzone R., Ferrari A., Cipullo I., Liberale V., D’Alonzo M., Maggiorotto F., Biglia N. (2019). Concerns and expectations of risk-reducing surgery in women with hereditary breast and ovarian cancer syndrome. J. Clin. Med..

[16] Narrandes S., Xu W. (2018). Gene expression detection assay for cancer clinical use. J. Cancer.

[17] Schiffman J.D., Fisher P.G., Gibbs P. (2015). Early detection of cancer: Past, present, and future. Am. Soc. Clin. Oncol. Educ. Book.

[18] Loud J.T., Murphy J. (2017). Cancer screening and early detection in the 21(st) century. Semin. Oncol. Nurs..

[19] Thomas L., Leiponen A. (2016). Big data commercialization. IEEE Eng. Manag. Rev..

[20] Wilusz C.J., Wilusz J. (2013). Lsm proteins and Hfq: Life at the 3′ end. RNA Biol..

[21] Reeves W.H., Narain S., Satoh M. (2003). Henry Kunkel, Stephanie Smith, clinical immunology, and split genes. Lupus.

[22] Li W., Li X., Gao L.N., You C.G. (2020). Integrated analysis of the functions and prognostic values of RNA binding proteins in lung squamous cell carcinoma. Front Genet..

[23] Streicher K.L., Yang Z.Q., Draghici S., Ethier S.P. (2007). Transforming function of the LSM1 oncogene in human breast cancers with the 8p11–12 amplicon. Oncogene.

[24] Watson P.M., Miller S.W., Fraig M., Cole D.J., Watson D.K., Boylan A.M. (2008). CaSm (LSm-1) overexpression in lung cancer and mesothelioma is required for transformed phenotypes. Am. J. Respir. Cell Mol. Biol..

[25] Luhtala N., Parker R. (2009). LSM1 over-expression in Saccharomyces cerevisiae depletes U6 snRNA levels. Nucleic Acids Res..

[26] Tao Y., Han Y., Yu L., Wang Q., Leng S.X., Zhang H. (2020). The predicted key molecules, functions, and pathways that bridge Mild Cognitive Impairment (MCI) and Alzheimer’s Disease (AD). Front Neurol..

[27] Brčić L., Barić A., Gračan S., Torlak V., Brekalo M., Škrabić V., Zemunik T., Barbalić M., Punda A., Boraska Perica V. (2019). Genome-wide association analysis suggests novel loci underlying thyroid antibodies in Hashimoto’s thyroiditis. Sci. Rep..

[28] Chong W., Shang L., Liu J., Fang Z., Du F., Wu H., Liu Y., Wang Z., Chen Y., Jia S. (2021). A regulator-based methylation modification patterns characterized by distinct tumor microenvironment immune profiles in colon cancer. Theranostics.

[29] Shahjaman M., Manir Hossain Mollah M., Rezanur Rahman M., Islam S.M.S., Nurul Haque Mollah M. (2020). Robust identification of differentially expressed genes from RNA-seq data. Genomics.

[30] Garcia-Reyero N., Griffitt R., Liu L., Kroll K., Farmerie W., Barber D., Denslow N. (2008). Construction of a robust microarray from a non-model species largemouth bass, Micropterus salmoides (Lacèpede), using pyrosequencing technology. J. Fish Biol..

[31] Llorens F., Hummel M., Pantano L., Pastor X., Vivancos A., Castillo E., Mattlin H., Ferrer A., Ingham M., Noguera M. (2013). Microarray and deep sequencing cross-platform analysis of the mirRNome and isomiR variation in response to epidermal growth factor. BMC Genom..

[32] Perez-Santángelo S., Mancini E., Francey L.J., Schlaen R.G., Chernomoretz A., Hogenesch J.B., Yanovsky M.J. (2014). Role for LSM genes in the regulation of circadian rhythms. Proc. Nat. Acad. Sci. USA.

[33] Derksen A., Shih H.-Y., Forget D., Darbelli L., Tran L.T., Poitras C., Guerrero K., Tharun S., Alkuraya F.S., Kurdi W.I. (2021). Variants in LSM7 impair LSM complexes assembly, neurodevelopment in zebrafish and may be associated with an ultra-rare neurological disease. Hum. Genet. Genom. Adv..

[34] Chandrashekar D.S., Bashel B., Balasubramanya S.A.H., Creighton C.J., Ponce-Rodriguez I., Chakravarthi B.V.S.K., Varambally S. (2017). UALCAN: A portal for facilitating tumor subgroup gene expression and survival analyses. Neoplasia.

[35] Modhukur V., Iljasenko T., Metsalu T., Lokk K., Laisk-Podar T., Vilo J. (2018). MethSurv: A web tool to perform multivariable survival analysis using DNA methylation data. Epigenomics.

[36] Curtis C., Shah S.P., Chin S.F., Turashvili G., Rueda O.M., Dunning M.J., Speed D., Lynch A.G., Samarajiwa S., Yuan Y. (2012). The genomic and transcriptomic architecture of 2000 breast tumours reveals novel subgroups. Nature.

[37] Koboldt D.C., Fulton R.S., McLellan M.D., Schmidt H., Kalicki-Veizer J., McMichael J.F., Fulton L.L., Dooling D.J., Ding L., Mardis E.R. (2012). Comprehensive molecular portraits of human breast tumours. Nature.

[38] Gao J., Aksoy B.A., Dogrusoz U., Dresdner G., Gross B., Sumer S.O., Sun Y., Jacobsen A., Sinha R., Larsson E. (2013). Integrative analysis of complex cancer genomics and clinical profiles using the cBioPortal. Sci. Sig..

[39] Network C.G.A. (2012). Comprehensive molecular portraits of human breast tumours. Nature.

[40] Hagerling C., Gonzalez H., Salari K., Wang C.-Y., Lin C., Robles I., van Gogh M., Dejmek A., Jirström K., Werb Z. (2019). Immune effector monocyte–neutrophil cooperation induced by the primary tumor prevents metastatic progression of breast cancer. Proc. Nat. Acad. Sci. USA.

[41] Wang C.-Y., Chiao C.-C., Phan N.N., Li C.-Y., Sun Z.-D., Jiang J.-Z., Hung J.-H., Chen Y.-L., Yen M.-C., Weng T.-Y. (2020). Gene signatures and potential therapeutic targets of amino acid metabolism in estrogen receptor-positive breast cancer. Am. J. Cancer Res..

[42] Gbenedio O.M., Bonnans C., Grun D., Wang C.-Y., Hatch A.J., Mahoney M.R., Barras D., Matli M., Miao Y., Garcia K.C. (2019). RasGRP1 is a potential biomarker for stratifying anti-EGFR therapy response in colorectal cancer. JCI Insight.

[43] Sun Z., Wang C.-Y., Lawson D.A., Kwek S., Velozo H.G., Owyong M., Lai M.-D., Fong L., Wilson M., Su H. (2018). Single-cell RNA sequencing reveals gene expression signatures of breast cancer-associated endothelial cells. Oncotarget.

[44] Wang C.-Y., Li C.-Y., Hsu H.-P., Cho C.-Y., Yen M.-C., Weng T.-Y., Chen W.-C., Hung Y.-H., Lee K.-T., Hung J.-H. (2017). PSMB5 plays a dual role in cancer development and immunosuppression. Am. J. Cancer Res..

[45] Chen P.S., Hsu H.P., Phan N.N., Yen M.C., Chen F.W., Liu Y.W., Lin F.P., Feng S.Y., Cheng T.L., Yeh P.H. (2021). CCDC167 as a potential therapeutic target and regulator of cell cycle-related networks in breast cancer. Aging.

[46] Lawson D.A., Bhakta N.R., Kessenbrock K., Prummel K.D., Yu Y., Takai K., Zhou A., Eyob H., Balakrishnan S., Wang C.-Y. (2015). Single-cell analysis reveals a stem-cell program in human metastatic breast cancer cells. Nature.

[47] Györffy B., Lanczky A., Eklund A.C., Denkert C., Budczies J., Li Q., Szallasi Z. (2010). An online survival analysis tool to rapidly assess the effect of 22,277 genes on breast cancer prognosis using microarray data of 1809 patients. Breast. Cancer Res. Treat..

[48] Thul P.J., Lindskog C. (2018). The human protein atlas: A spatial map of the human proteome. Protein Sci..

[49] Li T., Fan J., Wang B., Traugh N., Chen Q., Liu J.S., Li B., Liu X.S. (2017). TIMER: A Web server for comprehensive analysis of tumor-infiltrating immune cells. Cancer Res..

[50] Kao T.J., Wu C.C., Phan N.N., Liu Y.H., Ta H.D.K., Anuraga G., Wu Y.F., Lee K.H., Chuang J.Y., Wang C.Y. (2021). Prognoses and genomic analyses of proteasome 26S subunit, ATPase (PSMC) family genes in clinical breast cancer. Aging.

[51] Choy T.K., Wang C.Y., Phan N.N., Khoa Ta H.D., Anuraga G., Liu Y.H., Wu Y.F., Lee K.H., Chuang J.Y., Kao T.J. (2021). Identification of Dipeptidyl Peptidase (DPP) family genes in clinical breast cancer patients via an integrated bioinformatics approach. Diagnostics.

[52] Wu Y.H., Yeh I.J., Phan N.N., Yen M.C., Hung J.H., Chiao C.C., Chen C.F., Sun Z., Hsu H.P., Wang C.Y. (2021). Gene signatures and potential therapeutic targets of Middle East respiratory syndrome coronavirus (MERS-CoV)-infected human lung adenocarcinoma epithelial cells. J. Microbiol. Immunol. Infect..

[53] Anuraga G., Tang W.C., Phan N.N., Ta H.D.K., Liu Y.H., Wu Y.F., Lee K.H., Wang C.Y. (2021). Comprehensive Analysis of Prognostic and Genetic Signatures for General Transcription Factor III (GTF3) in clinical colorectal cancer patients using bioinformatics approaches. Curr. Issues Mol. Biol..

[54] Khoa Ta H.D., Tang W.C., Phan N.N., Anuraga G., Hou S.Y., Chiao C.C., Liu Y.H., Wu Y.F., Lee K.H., Wang C.Y. (2021). Analysis of LAGEs family gene signature and prognostic relevance in breast cancer. Diagnostics.

[55] Jin B., Li Y., Robertson K.D. (2011). DNA methylation: Superior or subordinate in the epigenetic hierarchy?. Genes Cancer.

[56] Daniel F.I., Cherubini K., Yurgel L.S., de Figueiredo M.A., Salum F.G. (2011). The role of epigenetic transcription repression and DNA methyltransferases in cancer. Cancer.

[57] Whiteside T.L. (2008). The tumor microenvironment and its role in promoting tumor growth. Oncogene.

[58] Wang M., Zhao J., Zhang L., Wei F., Lian Y., Wu Y., Gong Z., Zhang S., Zhou J., Cao K. (2017). Role of tumor microenvironment in tumorigenesis. J. Cancer.

[59] Ribeiro Franco P.I., Rodrigues A.P., de Menezes L.B., Pacheco Miguel M. (2020). Tumor microenvironment components: Allies of cancer progression. Pathol. Res. Pract..

[60] Joyce J.A. (2005). Therapeutic targeting of the tumor microenvironment. Cancer Cell.

[61] Bei Y., Cheng N., Chen T., Shu Y., Yang Y., Yang N., Zhou X., Liu B., Wei J., Liu Q. (2020). CDK5 Inhibition Abrogates TNBC Stem-Cell Property and Enhances Anti-PD-1 Therapy. Adv. Sci..

[62] Yu J.M., Sun W., Wang Z.H., Liang X., Hua F., Li K., Lv X.X., Zhang X.W., Liu Y.Y., Yu J.J. (2019). TRIB3 supports breast cancer stemness by suppressing FOXO1 degradation and enhancing SOX2 transcription. Nat. Commun..

[63] Lahouel K., Younes L., Danilova L., Giardiello F.M., Hruban R.H., Groopman J., Kinzler K.W., Vogelstein B., Geman D., Tomasetti C. (2020). Revisiting the tumorigenesis timeline with a data-driven generative model. Proc. Natl. Acad. Sci. USA.

[64] Bray F., Ferlay J., Soerjomataram I., Siegel R.L., Torre L.A., Jemal A. (2018). Global cancer statistics 2018: GLOBOCAN estimates of incidence and mortality worldwide for 36 cancers in 185 countries. CA Cancer J. Clin..

[65] Sung H., Ferlay J., Siegel R.L., Laversanne M., Soerjomataram I., Jemal A., Bray F. (2021). Global Cancer Statistics 2020: GLOBOCAN Estimates of incidence and mortality worldwide for 36 cancers in 185 countries. CA Cancer J. Clin..

[66] Siegel R.L., Miller K.D., Fuchs H.E., Jemal A. (2021). Cancer Statistics, 2021. CA Cancer J. Clin..

[67] Surveillance, Epidemiology, and End Results Program (2018). Cancer Stat Facts: Female Breast Cancer.

[68] Barrett T., Wilhite S.E., Ledoux P., Evangelista C., Kim I.F., Tomashevsky M., Marshall K.A., Phillippy K.H., Sherman P.M., Holko M. (2013). NCBI GEO: Archive for functional genomics data sets—Update. Nucleic Acids Res..

[69] Lin J.C., Liu T.P., Yang P.M. (2020). CDKN2A-inactivated pancreatic ductal adenocarcinoma exhibits therapeutic sensitivity to paclitaxel: A bioinformatics study. J. Clin. Med..

[70] Lin T.Y., Wang P.W., Huang C.H., Yang P.M., Pan T.L. (2020). Characterizing the relapse potential in different luminal subtypes of breast cancers with functional proteomics. Int. J. Mol. Sci..

[71] Liu L.W., Hsieh Y.Y., Yang P.M. (2020). Bioinformatics Data Mining Repurposes the JAK2 (Janus Kinase 2) Inhibitor fedratinib for treating pancreatic ductal adenocarcinoma by reversing the KRAS (Kirsten Rat Sarcoma 2 Viral Oncogene Homolog)-driven gene signature. J. Pers. Med..

[72] Yang P.M., Hsieh Y.Y., Du J.L., Yen S.C., Hung C.F. (2020). Sequential interferon β-cisplatin treatment enhances the surface exposure of calreticulin in cancer cells via an interferon regulatory factor 1-dependent manner. Biomolecules.

[73] Yang P.M., Lin L.S., Liu T.P. (2020). Sorafenib inhibits Ribonucleotide Reductase Regulatory Subunit M2 (RRM2) in hepatocellular carcinoma cells. Biomolecules.

[74] Kumar A., Swain C.A., Shevde L.A. (2021). Informing the new developments and future of cancer immunotherapy: Future of cancer immunotherapy. Cancer Metastasis Rev..

[75] Nelson M.A., Ngamcherdtrakul W., Luoh S.W., Yantasee W. (2021). Prognostic and therapeutic role of tumor-infiltrating lymphocyte subtypes in breast cancer. Cancer Metastasis Rev..

[76] Luo J., Liu S., Leung S., Gru A.A., Tao Y., Hoog J., Ho J., Davies S.R., Allred D.C., Salavaggione A.L. (2017). An mRNA gene expression-based signature to identify FGFR1-amplified estrogen receptor-positive breast tumors. J. Mol. Diagn..

[77] Young J.H., Peyton M., Seok Kim H., McMillan E., Minna J.D., White M.A., Marcotte E.M. (2016). Computational discovery of pathway-level genetic vulnerabilities in non-small-cell lung cancer. Bioinformatics.

[78] Rosen J., He M., Umbricht C., Alexander H.R., Dackiw A.P., Zeiger M.A., Libutti S.K. (2005). A six-gene model for differentiating benign from malignant thyroid tumors on the basis of gene expression. Surgery.

[79] Kontomanolis E.N., Koutras A., Syllaios A., Schizas D., Mastoraki A., Garmpis N., Diakosavvas M., Angelou K., Tsatsaris G., Pagkalos A. (2020). Role of oncogenes and tumor-suppressor genes in carcinogenesis: A review. Anticancer Res..

[80] Yang Z.Q., Streicher K.L., Ray M.E., Abrams J., Ethier S.P. (2006). Multiple interacting oncogenes on the 8p11–p12 amplicon in human breast cancer. Cancer Res..

[81] Yang Z.Q., Liu G., Bollig-Fischer A., Giroux C.N., Ethier S.P. (2010). Transforming properties of 8p11–12 amplified genes in human breast cancer. Cancer Res..

[82] Little E.C., Camp E.R., Wang C., Watson P.M., Watson D.K., Cole D.J. (2016). The CaSm (LSm1) oncogene promotes transformation, chemoresistance and metastasis of pancreatic cancer cells. Oncogenesis.

[83] Pan Y., Liu H., Wang Y., Kang X., Liu Z., Owzar K., Han Y., Su L., Wei Y., Hung R.J. (2017). Associations between genetic variants in mRNA splicing-related genes and risk of lung cancer: A pathway-based analysis from published GWASs. Sci. Rep..

[84] Lyng H., Brøvig R.S., Svendsrud D.H., Holm R., Kaalhus O., Knutstad K., Oksefjell H., Sundfør K., Kristensen G.B., Stokke T. (2006). Gene expressions and copy numbers associated with metastatic phenotypes of uterine cervical cancer. BMC Genom..

[85] Tan M.S., Chang S.W., Cheah P.L., Yap H.J. (2018). Integrative machine learning analysis of multiple gene expression profiles in cervical cancer. PeerJ..

[86] Xie N., Yao Y., Wan L., Zhu T., Liu L., Yuan J. (2017). Next-generation sequencing reveals lymph node metastasis associated genetic markers in colorectal cancer. Exp. Ther. Med..

[87] Gandini R., Dossena S., Vezzoli V., Tamplenizza M., Salvioni E., Ritter M., Paulmichl M., Furst J. (2008). LSm4 associates with the plasma membrane and acts as a co-factor in cell volume regulation. Cell Physiol. Biochem..

[88] Xue R., Hua L., Xu W., Gao Y., Pang Y., Hao J. (2018). Derivation and validation of the potential core genes in pancreatic cancer for tumor-stroma crosstalk. Biomed. Res. Int..

[89] Long Y., Weng W.H., Li F., Yuan X., Li Z. (2014). The effect of RNA binding protein Lsm4 on the proliferation and migration of esophageal carcinoma cell line EC109. Tumor.

[90] Hou W., Zhang Y. (2021). Circ_0025033 promotes the progression of ovarian cancer by activating the expression of LSM4 via targeting miR-184. Pathol. Res. Pract..

[91] Yin J., Lin C., Jiang M., Tang X., Xie D., Chen J., Ke R. (2021). CENPL, ISG20L2, LSM4, MRPL3 are four novel hub genes and may serve as diagnostic and prognostic markers in breast cancer. Sci. Rep..

[92] Wang L., Wrobel J.A., Xie L., Li D., Zurlo G., Shen H., Yang P., Wang Z., Peng Y., Gunawardena H.P. (2018). Novel RNA-affinity proteogenomics dissects tumor heterogeneity for revealing personalized markers in precision prognosis of cancer. Cell Chem. Biol..

[93] Kamikubo Y. (2018). Genetic compensation of RUNX family transcription factors in leukemia. Cancer Sci..

[94] Carelle-Calmels N., Saugier-Veber P., Girard-Lemaire F., Rudolf G., Doray B., Guérin E., Kuhn P., Arrivé M., Gilch C., Schmitt E. (2009). Genetic compensation in a human genomic disorder. N. Engl. J. Med..

[95] Tomlinson I.P.M., Novelli M.R., Bodmer W.F. (1996). The mutation rate and cancer. Proc. Nat. Acad. Sci. USA.

[96] Van Aelst L., D’Souza-Schorey C. (1997). Rho GTPases and signaling networks. Genes Dev..

[97] Wang L., Wang C., Su B., Song Q., Zhang Y., Luo Y., Li Q., Tan W., Ma D., Wang L. (2013). Recombinant human PDCD5 protein enhances chemosensitivity of breast cancer in vitro and in vivo. Biochem. Cell Biol..

[98] Zhang H., Zhang L.Q., Yang C.C., Li J., Tian X.Y., Li D.N., Cui J., Cai J.P. (2021). The high expression of NUDT5 indicates poor prognosis of breast cancer by modulating AKT/Cyclin D signaling. PLoS ONE.

[99] Van Genderen C., Harkness T.A.A., Arnason T.G. (2020). The role of anaphase promoting complex activation, inhibition and substrates in cancer development and progression. Aging.

[100] Chan S., Sridhar P., Kirchner R., Lock Y.J., Herbert Z., Buonamici S., Smith P., Lieberman J., Petrocca F. (2017). Basal-A Triple-Negative Breast Cancer Cells Selectively Rely on RNA Splicing for Survival. Mol. Cancer Ther..

[101] Tay R.E., Richardson E.K., Toh H.C. (2021). Revisiting the role of CD4+ T cells in cancer immunotherapy—New insights into old paradigms. Cancer Gene Ther..

[102] Chan M.-Y., Lee B.-J., Chang P.-S., Hsiao H.-Y., Hsu L.-P., Chang C.-H., Lin P.-T. (2020). The risks of ubiquinone and β-carotene deficiency and metabolic disorders in patients with oral cancer. BMC Cancer.

[103] Zhao L., Yang X., Feng C., Wang Y., Wang Q., Pei J., Wu J., Li S., Zhang H., Cao X. (2021). Triple-negative breast cancer cells respond to T cells severely at the alternative splicing layer. Electr. J. Biotechnol..

[104] Jaillon S., Ponzetta A., Di Mitri D., Santoni A., Bonecchi R., Mantovani A. (2020). Neutrophil diversity and plasticity in tumour progression and therapy. Nat. Rev. Cancer.

[105] Wu L., Saxena S., Awaji M., Singh R.K. (2019). Tumor-associated neutrophils in cancer: Going pro. Cancers.

[106] Olson M.F., Sahai E. (2008). The actin cytoskeleton in cancer cell motility. Clin. Exp. Metastasis.

[107] Yamaguchi H., Condeelis J. (2007). Regulation of the actin cytoskeleton in cancer cell migration and invasion. Biochim. Biophys. Acta (BBA) Mol. Cell Res..

[108] Ostuni R., Kratochvill F., Murray P.J., Natoli G. (2015). Macrophages and cancer: From mechanisms to therapeutic implications. Trends Immunol..

[109] Duan Z., Luo Y. (2021). Targeting macrophages in cancer immunotherapy. Sign. Transduct. Target. Ther..

[110] Nielsen S.R., Schmid M.C. (2017). Macrophages as key drivers of cancer progression and metastasis. Mediat. Inflamm..

[111] Binnewies M., Roberts E.W., Kersten K., Chan V., Fearon D.F., Merad M., Coussens L.M., Gabrilovich D.I., Ostrand-Rosenberg S., Hedrick C.C. (2018). Understanding the tumor immune microenvironment (TIME) for effective therapy. Nat. Med..

[112] Burugu S., Asleh-Aburaya K., Nielsen T.O. (2017). Immune infiltrates in the breast cancer microenvironment: Detection, characterization and clinical implication. Breast Cancer.

[113] Cohen I.J., Blasberg R. (2017). Impact of the tumor microenvironment on tumor-infiltrating lymphocytes: Focus on breast cancer. Breast Cancer.

[114] Luckheeram R.V., Zhou R., Verma A.D., Xia B. (2012). CD4⁺T cells: Differentiation and functions. Clin. Dev. Immunol..

[115] Ohue Y., Nishikawa H. (2019). Regulatory T (Treg) cells in cancer: Can Treg cells be a new therapeutic target?. Cancer Sci..

[116] Stenström J., Hedenfalk I., Hagerling C. (2021). Regulatory T lymphocyte infiltration in metastatic breast cancer—an independent prognostic factor that changes with tumor progression. Breast Cancer Res..

[117] Verma C., Kaewkangsadan V., Eremin J.M., Cowley G.P., Ilyas M., El-Sheemy M.A., Eremin O. (2015). Natural killer (NK) cell profiles in blood and tumour in women with large and locally advanced breast cancer (LLABC) and their contribution to a pathological complete response (PCR) in the tumour following neoadjuvant chemotherapy (NAC): Differential restoration of blood profiles by NAC and surgery. J. Transl. Med..

[118] Oh K., Lee O.Y., Shon S.Y., Nam O., Ryu P.M., Seo M.W., Lee D.S. (2013). A mutual activation loop between breast cancer cells and myeloid-derived suppressor cells facilitates spontaneous metastasis through IL-6 trans-signaling in a murine model. Breast Cancer Res..

[119] Markowitz J., Wesolowski R., Papenfuss T., Brooks T.R., Carson W.E. (2013). Myeloid-derived suppressor cells in breast cancer. Breast Cancer Res. Treat..

[120] Chen N., Feng Q., Deng J., Xiong Y., Deng Y.J., Wang M.M., Zhou L., Yu Q.W., Hu J.P., Deng H. (2020). Hdc-expressing myeloid-derived suppressor cells promote basal-like transition and metastasis of breast cancer. Int. J. Clin. Exp. Pathol..

[121] Xing S., Wang Y., Hu K., Wang F., Sun T., Li Q. (2020). WGCNA reveals key gene modules regulated by the combined treatment of colon cancer with PHY906 and CPT11. Biosci. Rep..

