# Supplementary material for: Potential Therapeutic and Prognostic Values of LSM Family Genes in Breast Cancer
Source: Cancers (Basel). 2021 Sep 29;13(19):4902. doi: 10.3390/cancers13194902 (PMC8508234; doi:10.3390/cancers13194902)
Supplement: Supplementary file 1 [file cancers-13-04902-s001.zip › cancers-1343149-supplementary.pdf]

Supplementary data

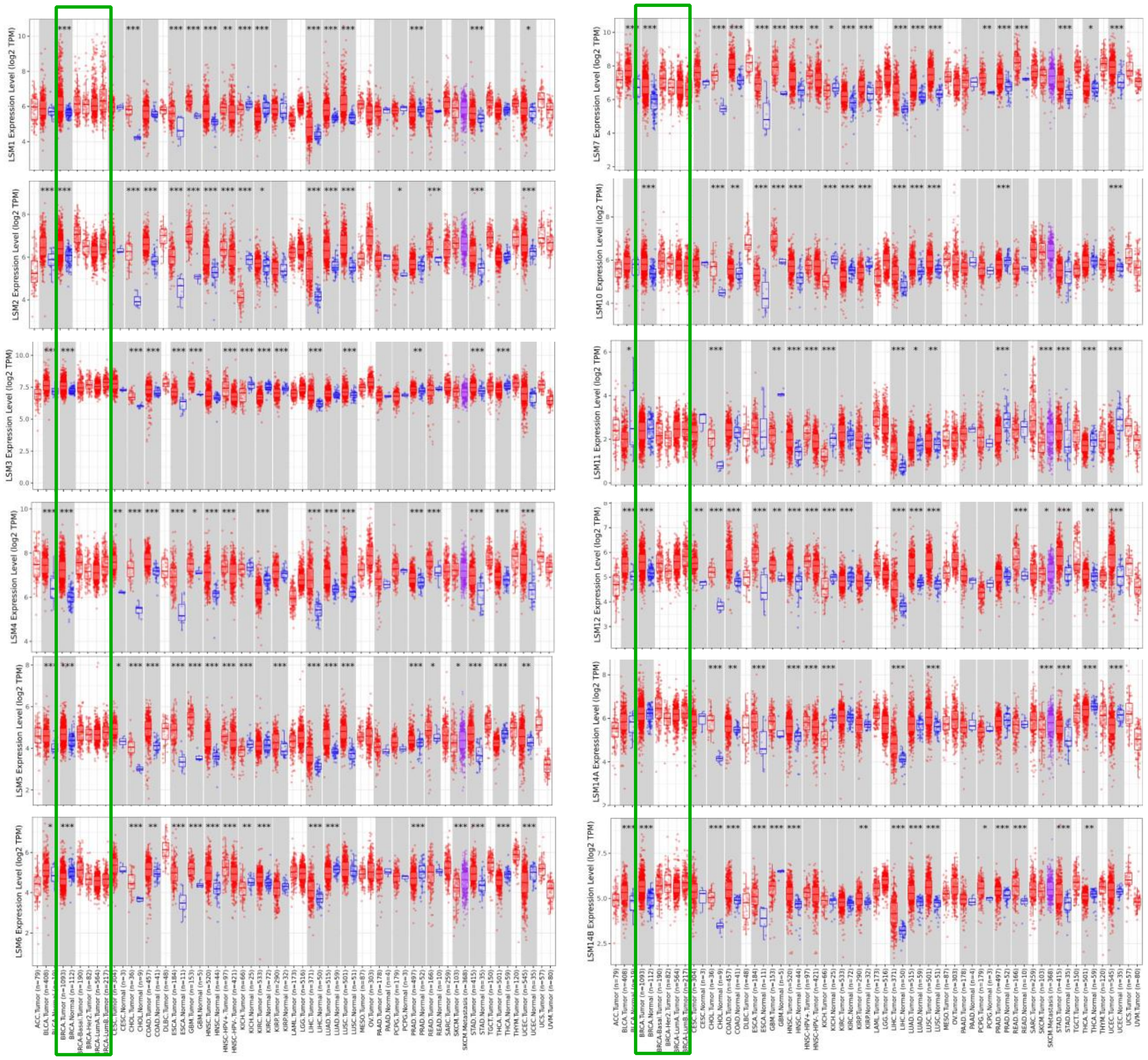

Figure S1. LSM family genes expression in several cancer types. Box plots display differential gene expression levels (Log2 TPM) of LSM family members between tumors and normal tissues from TCGA dataset. Blue boxes show normal samples, and red boxes indicate tumor tissues. Wilcoxon test was used to evaluate the statistical significance. * $p < 0.05$; ** $p < 0.01$; *** $p < 0.001$

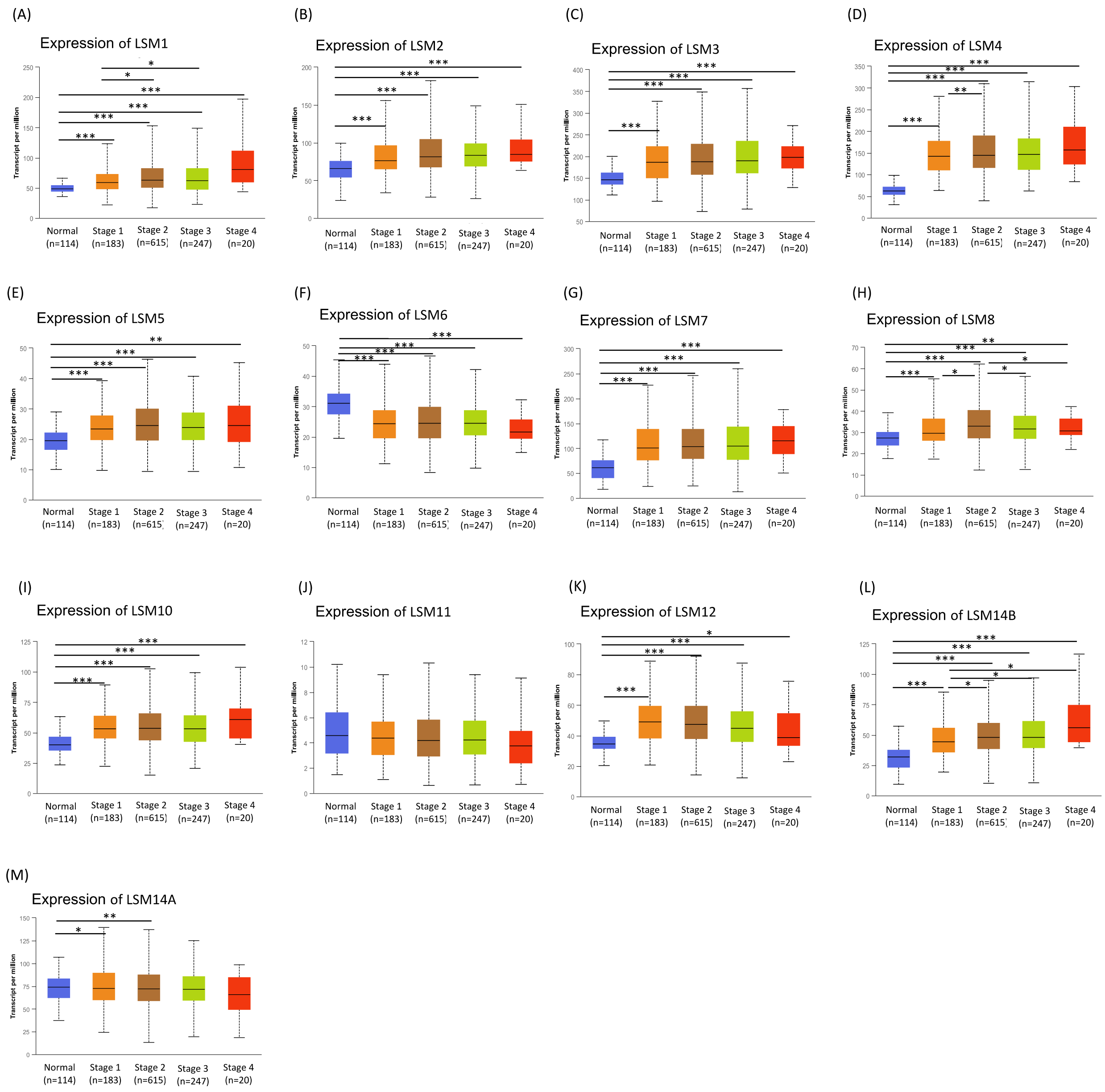

Figure S2. LSMs transcription levels in different stages of breast cancer (UALCAN database). In the TCGA dataset, there are 114 normal samples, 183 patients at Stage 1, 615 patients at Stage 2, 247 patients at Stage 3, 20 patients at Stage 4. Boxplots showing the relative expression levels of LSM family genes in normal samples and stage 1, 2, 3, 4 of breast cancer. Figures (A)-(M) depicted the expression of LSM family genes (LSM1-LSM14B) in breast cancer patients on individual cancer stages, respectively. Student's t-test was conducted for statistical tests between groups. *P < 0.05; **P < 0.01; ***P < 0.001.

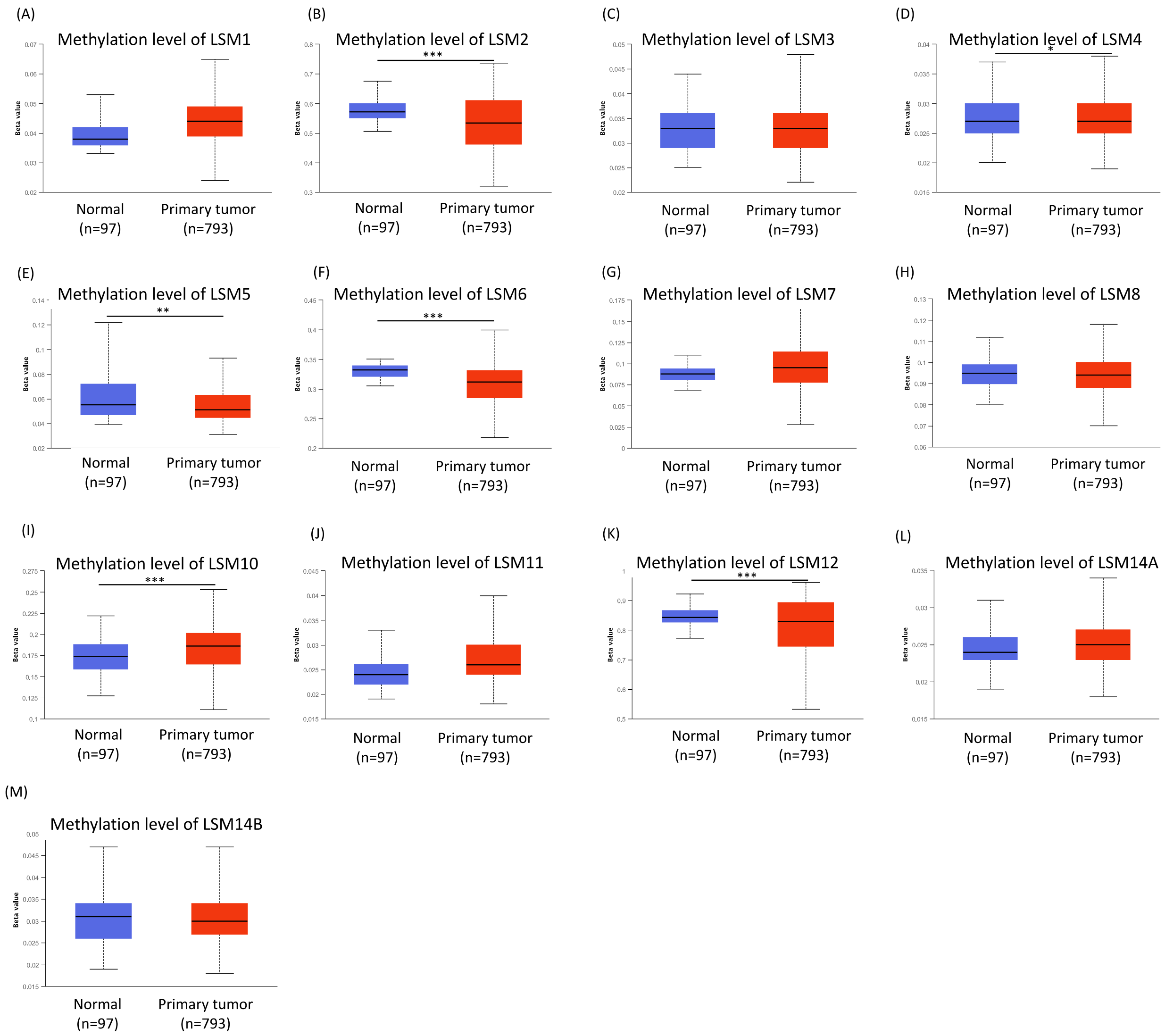

Figure S3. LSMs DNA methylation level in patients with breast cancer versus healthy controls (UALCAN database, with 97 normal samples and 793 primary tumor samples). P-value <0.05 was considered statistically significant by applying Student's t-test, which showed in red labels. (A)-(M) represented the comparison of methylation level between the normal sample and primary tumor of LSM1-LSM14B, respectively. *P < 0.05; **P < 0.01; ***P < 0.001.

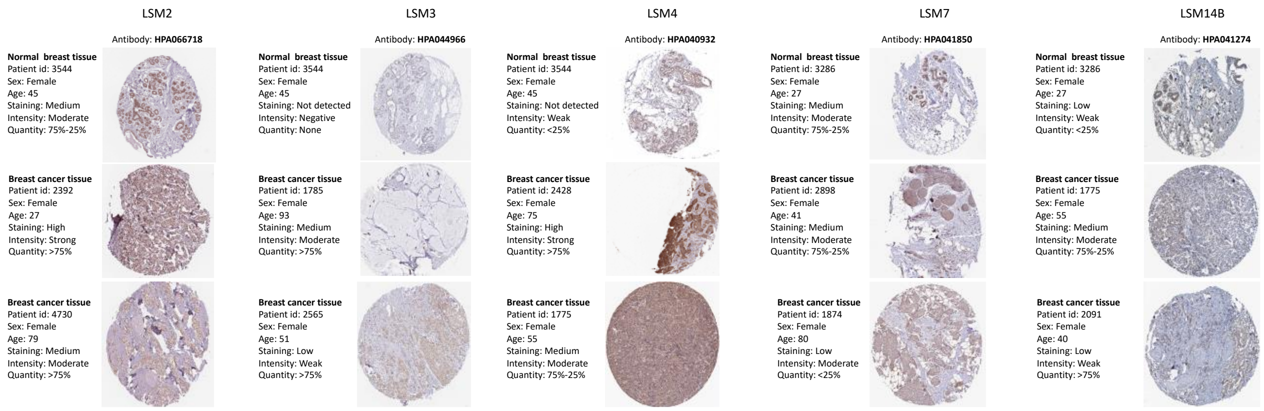

Figure S4. Representative immunohistochemistry images of LSM2, LSM3, LSM4, LSM7, LSM14b in breast cancer patients, which showed the normal and tumor samples (Human Protein Atlas), and IHC intensity of these genes as well, respectively. All the IHC images and patient information were obtained from Human Protein Atlas.

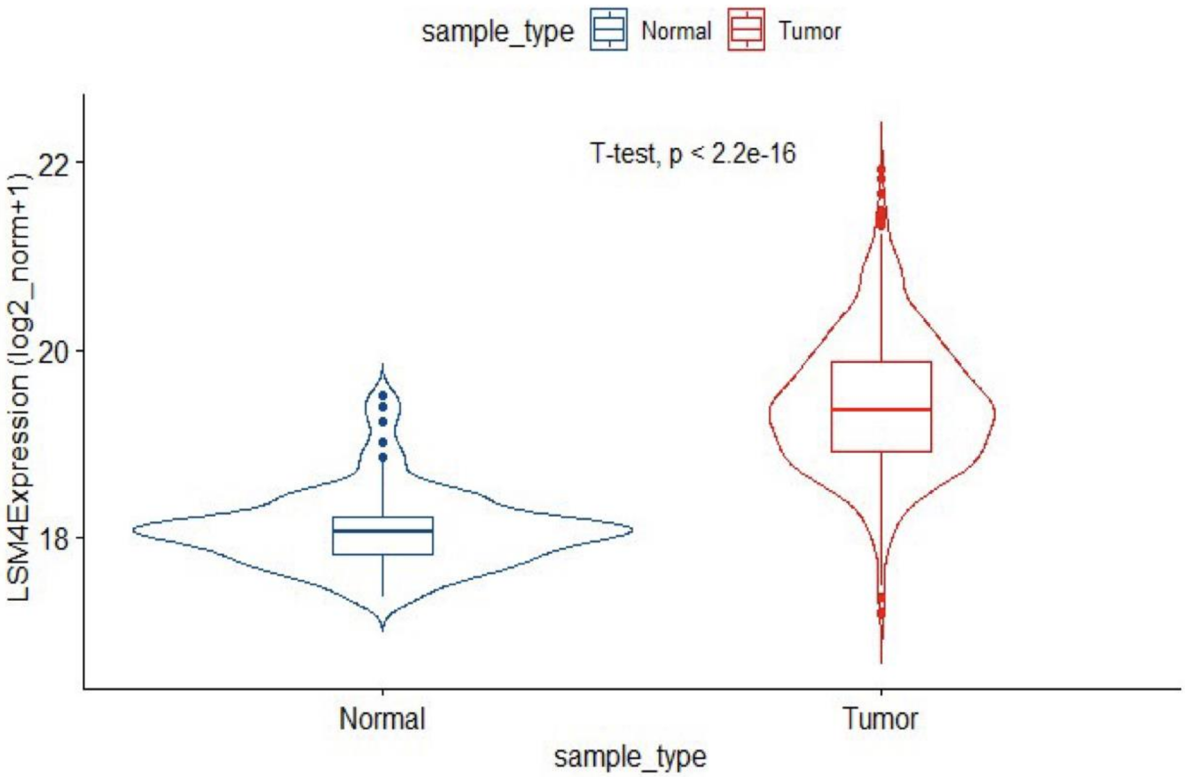

Figure S5. LSM4 expression between normal and tumor groups (n=1222 patients from GDC database). Violin plot shows LSM4 expression in both normal and tumor samples. A student's t-test was used and a p-value <0.05 was considered statistically significant.

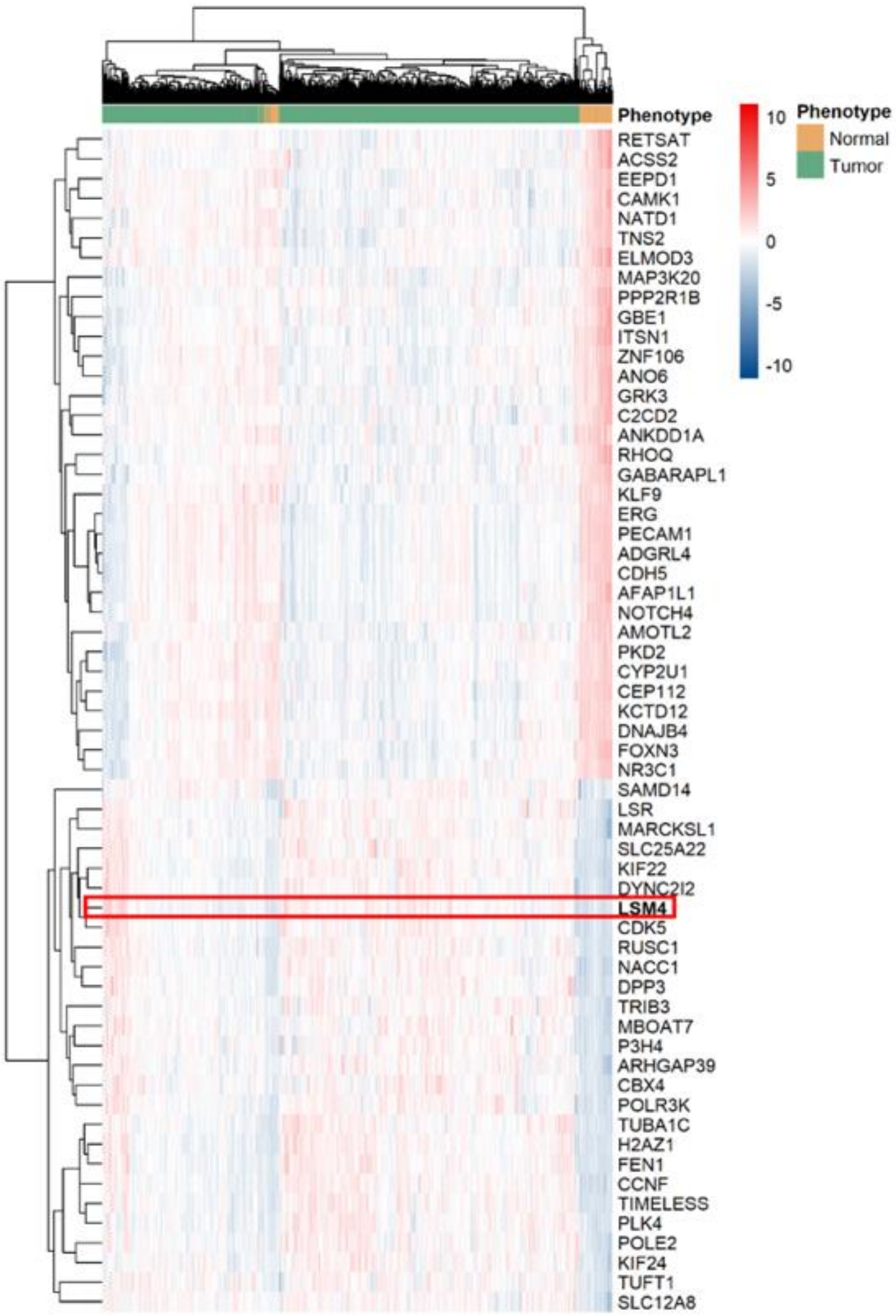

Figure S6. Expression profiles of LSM4 in DEGs methods. The platform GPL570 from the GDC dataset consists of 1222 samples of breast cancer patients and 18217 genome data in the form of genes was obtained to plot the Heatmap of top 60 genes based on log2foldchange values. P-values <0.05 and log2FC > 1.5 were set as threshold.

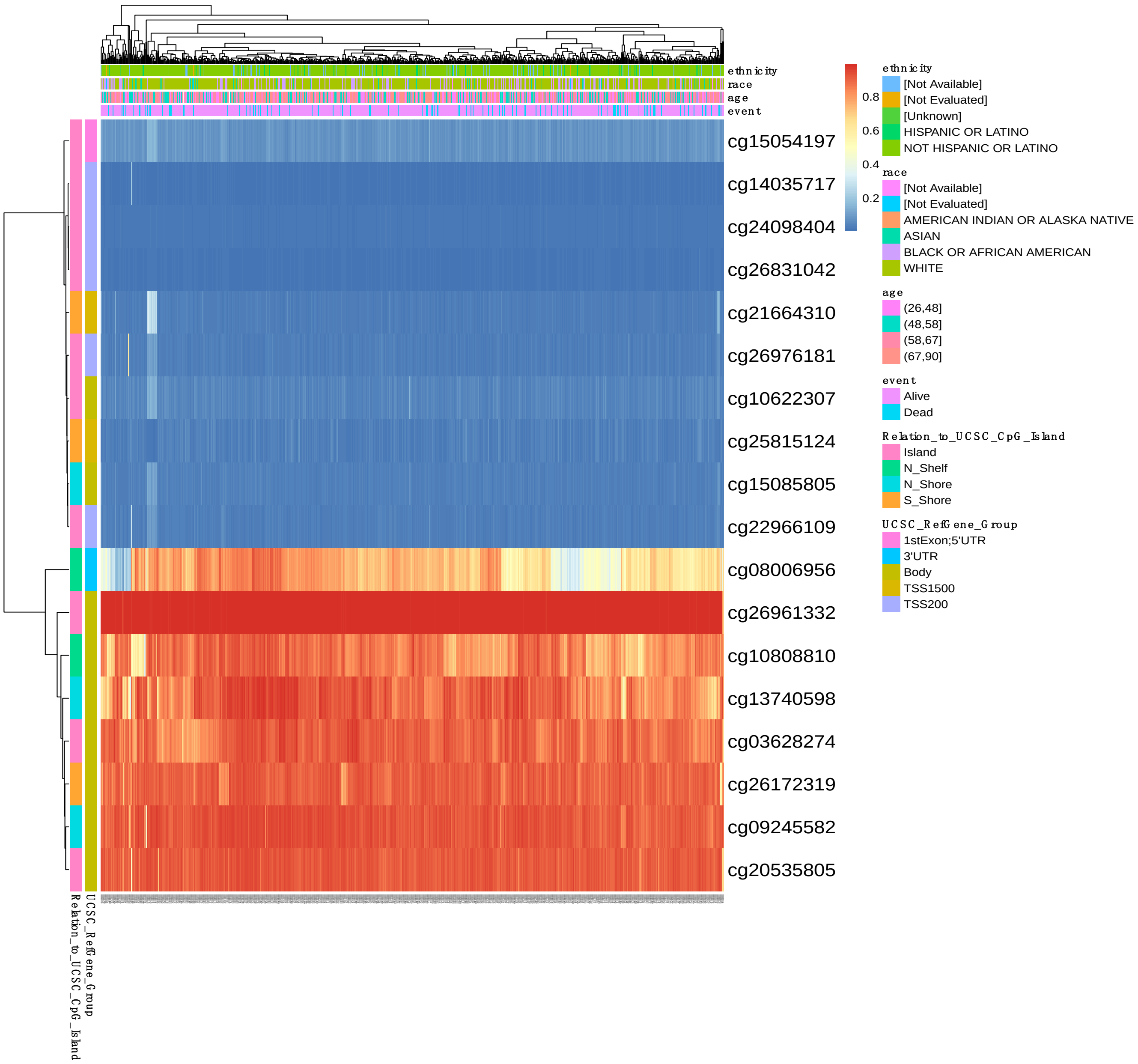

Figure S7. DNA methylation clustered expression of LSM4 (TCGA dataset). Blue bars indicate low expression, while red bars mean high expression. The different color side boxes denoted ethnicity, race, age, event, Relation to UCSC CpG island and UCSC RefGene Group. DNA methylation status was represented as β-values (ranging from 0 to 1).

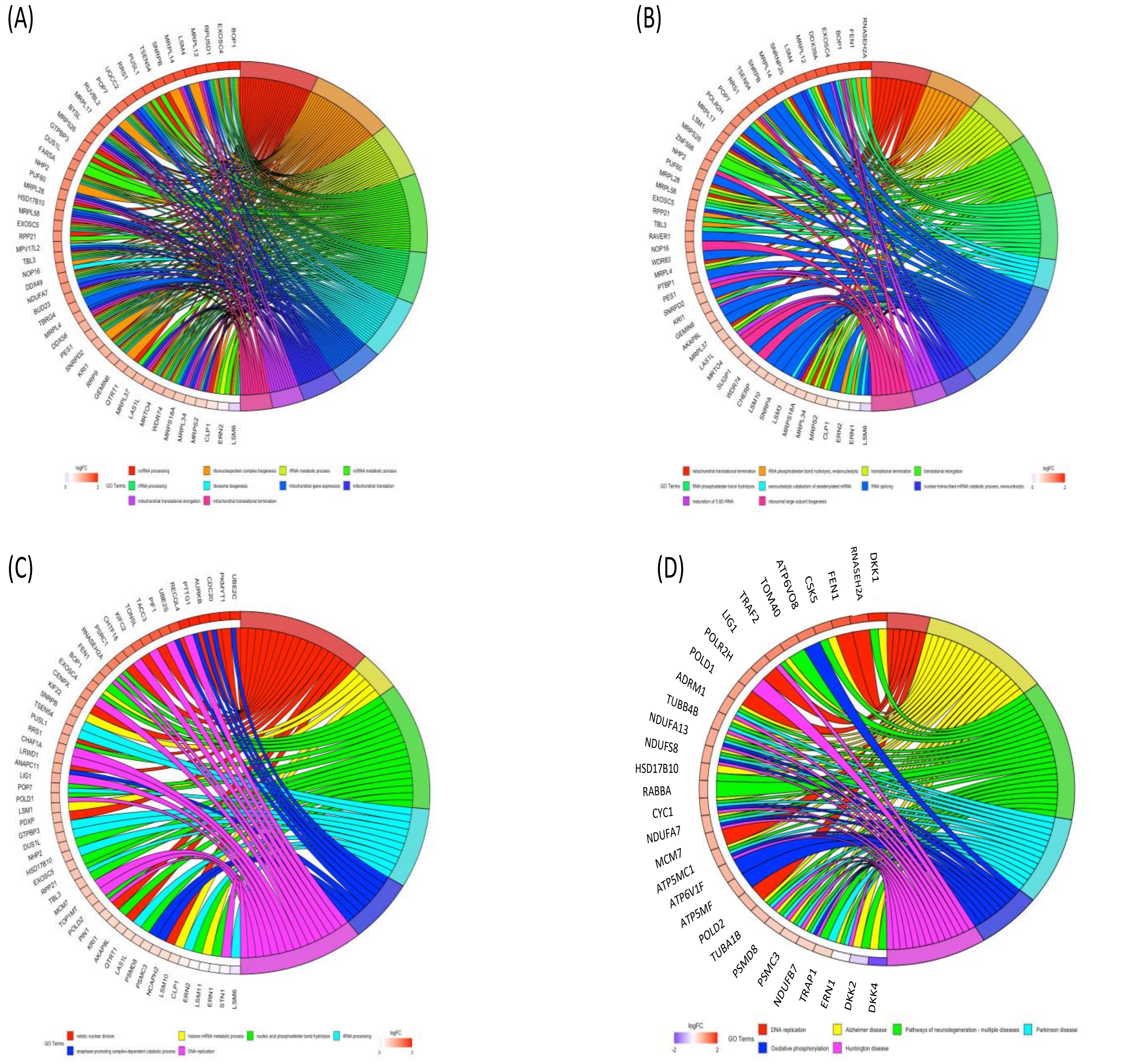

Figure S8. Functional enrichment analysis with gene ontology (GO) terms and KEGG of differentially expressed genes (DEGs). (A-C) Chord diagrams for biological processes, cellular components, and molecular functions, respectively. (D) KEGG terms of DEGs. The involved DEGs are shown on the left hand side of the chord plots. The red gene bars indicate upregulation, and blue ones stand for downregulation

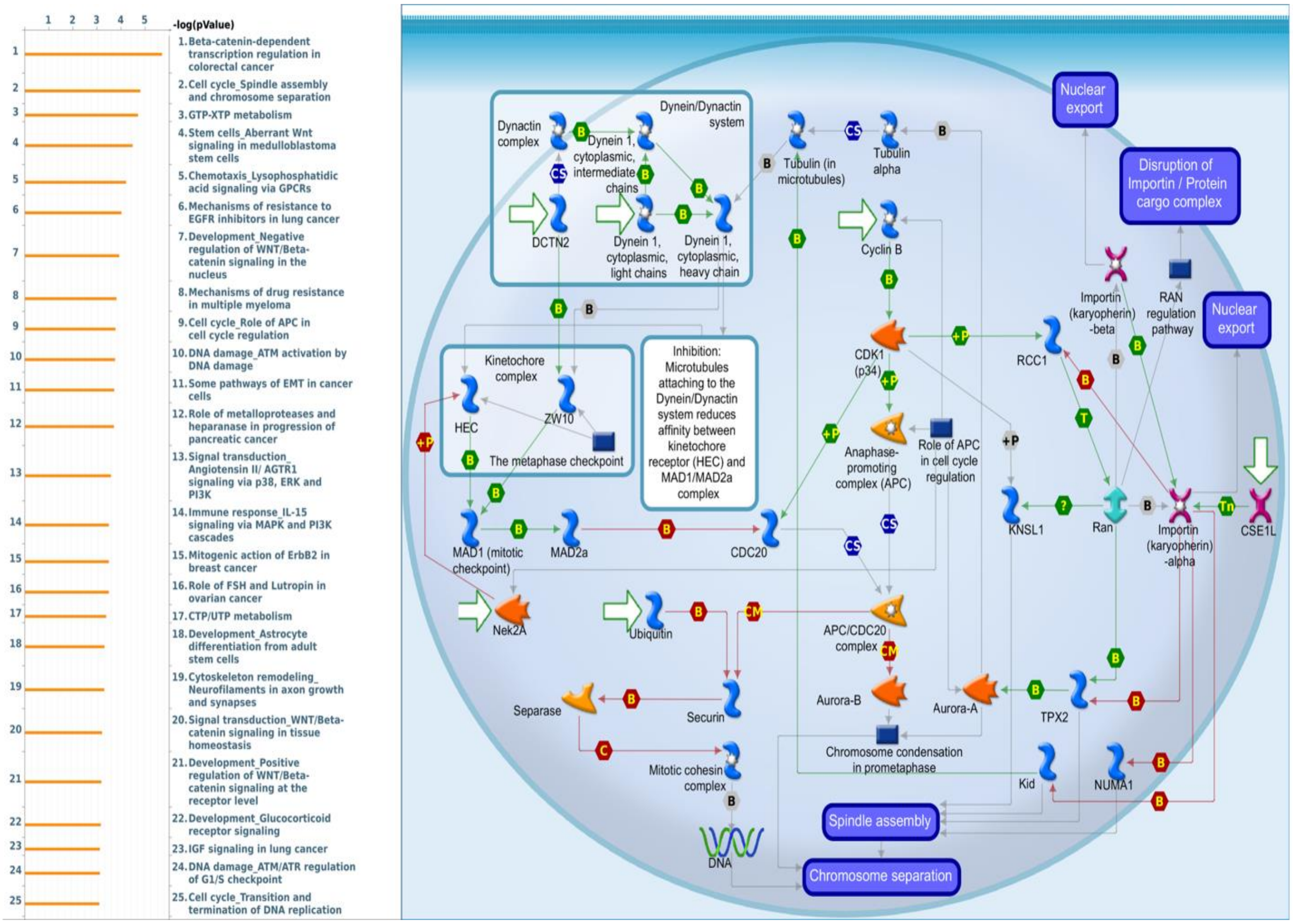

Figure S9. The approach was to collect the top 10% of expression gene lists from both METABRIC (2000 genes) and Pan-Cancer (1800 genes), then overlapping to a final genes list of 463 genes in total. The pathway list was ordered by the -log P-value from the genes list extracted via TCGA Pan-Cancer atlas and METABRIC breast cancer patient databases. “Cell cycle Spindle assembly and chromosome separation” placed on top of the pathway list when performing the “biological process” analysis

Table S1. The basic characteristic of LSM4 gene on Oncomine database

Gene	*Dataset*	*group comparison*	*fold change*	*p-value*	*number of patients*
LSM4	Curtis Breast (n=2136)	Invasive Ductal and Invasive Lobular Breast Carcinoma vs. Normal	2,111	2.64E-43	90/234
		Mucinous Breast Carcinoma vs. Normal	2,15	2.17E-23	46/190
		Invasive Ductal Breast Carcinoma vs. Normal	2,31	8.25E-91	1556/1700
		Breast Carcinoma vs. Normal	2,05	1.14E-08	14/158
		Medullary Breast Carcinoma vs. Normal	2,391	2.61E-14	32/176
	TCGA breast (n=593)	Invasive Ductal Breast Carcinoma vs. Normal	2,141	1,02E-38	389/450
	Richardson Breast 2 (n=47)	Ductal Breast Carcinoma vs. Normal	2,283	2.51E-08	40/47
	Ma breast 4 (n=66)	Lobular Breast Carcinoma vs. Normal	2,045	2.66E-07	9/23

Table S2. Statistical values of LSM family genes expression base on individual cancer stages

Comparison	P-value												
	LSM1	LSM2	LSM3	LSM4	LSM5	LSM6	LSM7	LSM8	LSM10	LSM11	LSM12	LSM14A	LSM14B
Normal - Stage 1	2,79E-08	1,97E-12	3,33E-16	1,62E-12	2,48E-05	1,47E-09	1,62E-12	1,19E-07	1,62E-12	2,40E-01	<1E-12	2,30E-02	1,62E-12
Normal - Stage 2	2,22E-16	1,62E-12	1,62E-12	1,62E-12	1,62E-12	2,13E-09	<1E-12	1,62E-12	1,62E-12	1,78E-01	<1E-12	1,22E-03	<1E-12
Normal - Stage 3	1,00E-11	1,62E-12	1,62E-12	1,62E-12	1,68E-12	1,91E-12	<1E-12	2,73E-11	3,33E-16	2,81E-01	1,11E-16	3,18E-01	1,62E-12
Normal - Stage 4	5,03E-04	4,16E-04	1,05E-04	9,86E-07	9,04E-03	7,23E-04	1,69E-05	1,54E-03	1,79E-04	2,01E-01	2,85E-02	6,44E-01	2,26E-06
Stage 1 - Stage 2	1,48E-02	3,30E-03	2,54E-03	7,80E-03	9,28E-01	2,22E-01	3,01E-01	4,38E-02	2,92E-01	9,58E-01	8,56E-01	9,91E-01	2,60E-03
Stage 1 - Stage 3	4,48E-02	3,68E-01	5,14E-03	1,13E-01	4,19E-01	8,90E-01	2,89E-01	9,97E-01	6,04E-01	8,83E-01	4,36E-02	1,60E-01	5,26E-03
Stage 1 - Stage 4	2,90E-01	2,33E-01	7,06E-01	1,75E-01	9,62E-01	8,56E-01	2,29E-01	5,82E-01	4,86E-02	4,67E-01	7,66E-02	2,03E-01	2,78E-03
Stage 2 - Stage 3	9,49E-01	1,99E-02	5,92E-01	4,29E-01	4,99E-02	8,54E-02	8,31E-01	1,95E-02	7,63E-01	8,08E-01	6,26E-03	5,45E-02	8,31E-01
Stage 2 - Stage 4	6,95E-01	9,70E-01	2,89E-01	7,46E-01	9,97E-01	5,62E-01	4,16E-01	2,56E-02	1,92E-01	4,81E-01	8,93E-02	1,89E-01	2,84E-01
Stage 3 - Stage 4	6,90E-01	3,62E-01	2,09E-01	5,51E-01	4,58E-01	8,75E-01	4,71E-01	5,50E-01	2,27E-01	4,39E-01	3,34E-01	3,89E-01	2,40E-01

Table S3. Prognosis value of CpGs in LSM4 (MethSurv database)

Name	Cancer	HR	CI	P.value	Best_split	MAPINFO	UCSC_RefGene_Name	UCSC_RefGene_Group	Relation_to_UCSC_CpG_Island
cg03628274	BRCA	1.151	(0.727-1.822)	0.54869470772557	q25	18420511	LSM4	Body	Island
cg08006956	BRCA	0.718	(0.444-1.162)	0.17766823143342	q75	18418056	LSM4	3'UTR	N_Shelf
cg09245582	BRCA	0.765	(0.518-1.131)	0.179847910094958	median	18419467	LSM4	Body	N_Shore
cg10622307	BRCA	1.166	(0.768-1.771)	0.470449265760582	q75	18433643	LSM4	Body	Island
cg10808810	BRCA	0.614	(0.375-1.004)	0.0520933372972706	q75	18429665	LSM4	Body	N_Shelf
cg13740598	BRCA	0.595	(0.4-0.885)	0.0103268375374075	median	18419211	LSM4	Body	N_Shore
cg14035717	BRCA	0.772	(0.512-1.165)	0.218227334938768	mean	18434138	LSM4	TSS200	Island
cg15054197	BRCA	0.763	(0.512-1.139)	0.1860680436322	mean	18433911	LSM4	1stExon;5'UTR	Island
cg15085805	BRCA	0.661	(0.415-1.052)	0.080779217494177	q25	18433592	LSM4	Body	N_Shore
cg20535805	BRCA	0.672	(0.454-0.994)	0.0465587163613179	mean	18420735	LSM4	Body	Island
cg21664310	BRCA	1.148	(0.753-1.751)	0.521058598421183	q75	18434339	LSM4	TSS1500	S_Shore
cg22966109	BRCA	1.472	(0.988-2.194)	0.0572423874442253	mean	18434177	LSM4	TSS200	Island
cg24098404	BRCA	1.301	(0.877-1.929)	0.19108487752662	median	18434142	LSM4	TSS200	Island
cg25815124	BRCA	0.853	(0.539-1.351)	0.498113218997267	q25	18434238	LSM4	TSS1500	S_Shore
cg26172319	BRCA	1.09	(0.736-1.615)	0.666996595435314	median	18421854	LSM4	Body	S_Shore
cg26831042	BRCA	0.629	(0.378-1.047)	0.0744852943406927	q75	18434038	LSM4	TSS200	Island
cg26961332	BRCA	0.564	(0.379-0.84)	0.00480201118681822	median	18420631	LSM4	Body	Island
cg26976181	BRCA	1.396	(0.864-2.257)	0.172953747405131	q25	18434160	LSM4	TSS200	Island

MAPINFO represents chromosome and position information; pvalue is calculated through the Wilcoxon rank-sum test followed by FDR (false discovery rate) adjustment for multiple corrections.

Table S4. Gene sets enriched in high expression LSM4

NAME	SIZE	ES	NES	NOM p-val	FDR q-val	FWER p-val	RANK AT MAX
HALLMARK_EPITHELIAL_MESENCHYMAL_TRANSITION	184	0.55581105	36.345.425	0.0	0.0	0.0	3918
HALLMARK_UV_RESPONSE_DN	130	0.46894485	29.184.568	0.0	0.0	0.0	4609
HALLMARK_KRAS_SIGNALING_UP	178	0.37147906	2.444.795	0.0	0.0	0.0	3930
HALLMARK_MYOGENESIS	190	0.32343954	213.766	0.0	4,46E+03	0.002	3887
HALLMARK_COAGULATION	126	0.32323095	19.785.095	0.0	0.0015085542	0.007	3890
HALLMARK_TGF_BETA_SIGNALING	52	0.38005242	19.463.059	0.0	0.0014065536	0.008	4190
HALLMARK_TNFA_SIGNALING_VIA_NFKB	185	0.27296796	18.122.653	0.0	0.0031423587	0.021	4705
HALLMARK_ANGIOGENESIS	35	0.3844226	17.665.083	0.0088495575	0.0050957585	0.04	4443
HALLMARK_ESTROGEN_RESPONSE_EARLY	179	0.2626482	17.251.546	0.0	0.0073388377	0.063	5058
HALLMARK_APICAL_JUNCTION	181	0.25376558	16.609.256	0.0	0.011154205	0.105	4688
HALLMARK_ANDROGEN_RESPONSE	88	0.2790822	15.872.321	0.006329114	0.01916953	0.185	4020
HALLMARK_WNT_BETA_CATENIN_SIGNALING	41	0.33050233	15.634.063	0.039697543	0.021157784	0.218	3994
HALLMARK_NOTCH_SIGNALING	30	0.34037733	14.941.674	0.046092186	0.036435455	0.347	4222
HALLMARK_IL2_STAT5_SIGNALING	176	0.22726999	14.850.502	0.013245033	0.03708512	0.379	5227
HALLMARK_INFLAMMATORY_RESPONSE	188	0.22321622	14.768.684	0.01934236	0.03659096	0.399	3883
HALLMARK_HEDGEHOG_SIGNALING	32	0.33398	14.532.586	0.06720977	0.039944455	0.451	4571
HALLMARK_ESTROGEN_RESPONSE_LATE	187	0.18929738	12.537.602	0.07114624	0.1604038	0.937	4872
HALLMARK_P53_PATHWAY	177	0.18405075	12.002.507	0.13097712	0.21183962	0.978	5146
HALLMARK_APICAL_SURFACE	38	0.2510013	11.657.314	0.2371134	0.25003025	0.993	3104
HALLMARK_HYPOXIA	176	0.16956826	1.106.618	0.23540856	0.33509856	0.998	5323
HALLMARK_XENOBIOTIC_METABOLISM	187	0.16728139	11.008.854	0.252505	0.3304838	0.998	5129
HALLMARK_APOPTOSIS	152	0.16951863	10.851.682	0.26104417	0.34244183	0.998	4398
HALLMARK_PANCREAS_BETA_CELLS	38	0.22809084	10.713.276	0.33333334	0.35183182	0.999	4196
HALLMARK_COMPLEMENT	184	0.15990062	10.614.573	0.30103093	0.356137	0.999	5461
HALLMARK_ALLOGRAFT_REJECTION	180	0.15927535	1.040.058	0.34631148	0.38013163	1.0	5238
HALLMARK_KRAS_SIGNALING_DN	171	0.14032325	0.90302485	0.6806723	0.65641874	1.0	4386

Table S5. Pathway analysis of LSM4-coexpressed genes from public breast cancer databases using the MetaCore database (p<0.01 set as the cutoff value)

#	Maps	pValue	Network Objects from Active Data
1	Beta-catenin-dependent transcription regulation in colorectal cancer	2,027E-06	CD44, ELAVL1 (HuR), CD44 soluble, TCF7L2 (TCF4), MDR1, YAP1 (YAp65), CD44 (EXT)
2	Cell cycle_Spindle assembly and chromosome separation	1,687E-05	Aurora-B, Kid, CDC20, Tubulin alpha, Securin, Tubulin (in microtubules)
3	GTP-XTP metabolism	2,130E-05	POLR2J, NDPK A, RRP41, RPOM, RRP46, RPB8, IMD1, RPA16, RPA39
4	Stem cells_Aberrant Wnt signaling in medulloblastoma stem cells	3,532E-05	CD44, DAB2, Axin1, Axin, TCF7L2 (TCF4)
5	Chemotaxis_Lysophosphatidic acid signaling via GPCRs	6,842E-05	LPAR2, FKHR, 4E-BP1, MEK1/2, Tcf(Lef), Rho GTPase, H-Ras, YAP1 (YAp65), G-protein gamma 12, PRK1

6	Mechanisms of resistance to EGFR inhibitors in lung cancer	1,047E-04	HSP90, 4E-BP1, TCF8, H-Ras, Survivin, MEK2(MAP2K2)
7	Development_Negative regulation of WNT/Beta-catenin signaling in the nucleus	1,316E-04	NARF, PJA2, RUVBL2, Tcf(Lef), Axin, TCF7L2 (TCF4), Menin, Histone H1
8	Mechanisms of drug resistance in multiple myeloma	1,699E-04	CD44, MEK1/2, GCR Alpha, H-Ras, TCF7L2 (TCF4), MDR1
9	Cell cycle_Role of APC in cell cycle regulation	1,867E-04	Tome-1, Aurora-B, Kid, CDC20, Securin
10	DNA damage_ATM activation by DNA damage	1,953E-04	HSP90, OBFC2B, TELO2, RecQL4, Histone H2AX, PP2A regulatory, CDK5
11	Some pathways of EMT in cancer cells	2,128E-04	JAK1, 4E-BP1, TRAF2, Axin, H-Ras, PDGF-D
12	Role of metalloproteases and heparanase in progression of pancreatic cancer	2,170E-04	CD44, CD44 soluble, H-Ras, MEK2(MAP2K2), CD44 (EXT)
13	Signal transduction_Angiotensin II/ AGTR1 signaling via p38, ERK and PI3K	2,958E-04	ELAVL1 (HuR), FKHR, 4E-BP1, MEK1/2, p70 S6 kinases, RECK, H-Ras, PDGF-D
14	Immune response_IL-15 signaling via MAPK and PI3K cascades	3,575E-04	JAK1, FKHR, MEK1/2, TRAF2, p70 S6 kinases, H-Ras
15	Mitogenic action of ErbB2 in breast cancer	3,575E-04	FKHR, MEK1/2, p70 S6 kinase2, H-Ras, NUMB, TCF7L2 (TCF4)
16	Role of FSH and Lutropin in ovarian cancer	3,575E-04	CD44, FKHR, MEK1/2, SLIT2, Survivin, ITGAV
17	CTP/UTP metabolism	4,683E-04	POLR2J, NDPK A, RRP41, RPOM, RRP46, RPB8, RPA16, RPA39
18	Development_Astrocyte differentiation from adult stem cells	5,464E-04	JAK1, CD44, MEK1/2, H-Ras, BMP receptor 2
19	Cytoskeleton remodeling_Neurofilaments in axon growth and synapses	5,555E-04	MEK1/2, BPAG1, CDK5, Tubulin (in microtubules)
20	Signal transduction_WNT/Beta-catenin signaling in tissue homeostasis	6,872E-04	FKHR, Tcf(Lef), TCF7L2 (TCF4), Survivin, Versican

21	Development_Positive regulation of WNT/Beta-catenin signaling at the receptor level	7,377E-04	CD44, ELAVL1 (HuR), Rab8B, MEK1/2, Tcf(Lef), RECK
22	Development_Glucocorticoid receptor signaling	7,727E-04	HSP90, GCR, GCR Alpha, GCR Beta
23	IGF signaling in lung cancer	8,535E-04	4E-BP1, Histone H2AX, H-Ras, Survivin, MEK2(MAP2K2)
24	DNA damage_ATM/ATR regulation of G1/S checkpoint	8,535E-04	ELAVL1 (HuR), Histone H2AX, PP2A regulatory, p70 S6 kinases, MEK2(MAP2K2)
25	Cell cycle_Transition and termination of DNA replication	9,011E-04	POLD reg (p50), FEN1, POLD cat (p125), DNA ligase I
26	Transcription_Negative regulation of HIF1A function	1,100E-03	HSP90, RUVBL2, Sirtuin7, MCM7, Elongin C, Elongin B
27	Canonical Leptin pathways in breast cancer	1,157E-03	MEK1/2, Tcf(Lef), Axin, H-Ras, Survivin
28	Canonical Notch signaling pathway in colorectal cancer	1,157E-03	CD44, 4E-BP1, NUMB, TCF7L2 (TCF4), Survivin
29	ATP/ITP metabolism	1,175E-03	POLR2J, NDPK A, RRP41, RPOM, RRP46, RPB8, RPA16, RPA39
30	Stellate cells activation and liver fibrosis	1,186E-03	DAB2, TRAF2, Tcf(Lef), TGF-beta receptor type II, H-Ras, MEK2(MAP2K2)
31	Histone deacetylases in Prostate Cancer	1,376E-03	HSP90, FKHR, Sirtuin7, Tubulin alpha
32	Immune response_IL-2 signaling via ERK, PI3K, and PLC-gamma	1,476E-03	JAK1, 4E-BP1, MEK1/2, p70 S6 kinase2, p70 S6 kinases, H-Ras
33	Immune response_BAFF-induced signaling	1,677E-03	FKHR, 4E-BP1, MEK1/2, TRAF2, TRIM2
34	Inflammatory factors-induced expression of mucins in normal and asthmatic epithelium	1,677E-03	JAK1, H-Ras, SF4, MEK2(MAP2K2), PGES2
35	NETosis in SLE	1,775E-03	Histone H2, Histone H2A, Pin1, Histone H1
36	IL-6 signaling in breast cancer cells	1,994E-03	JAK1, MEK1/2, H-Ras, MDR1, Survivin

37	Translation_Translation regulation by Alpha-1 adrenergic receptors	1,994E-03	4E-BP1, p70 S6 kinase2, H-Ras, MEK2(MAP2K2), PRK1
38	Cell cycle_Role of Nek in cell cycle regulation	2,002E-03	Tubulin beta, Tubulin alpha, Histone H1, Tubulin (in microtubules)
39	Cytoskeleton remodeling_Reverse signaling by Ephrin-B	2,002E-03	Axin, H-Ras, Tubulin alpha, Tubulin (in microtubules)
40	Translation_Regulation of EIF4F activity	2,167E-03	4E-BP1, p70 S6 kinase2, TGF-beta receptor type II, H-Ras, MEK2(MAP2K2)
41	Development_HGF-dependent inhibition of TGF-beta-induced EMT	2,514E-03	TGF-beta receptor type II, H-Ras, MEK2(MAP2K2), PGES2
42	Development_S1P1 receptor signaling via beta-arrestin	2,514E-03	p70 S6 kinase2, p70 S6 kinases, H-Ras, MEK2(MAP2K2)
43	Development_CNTF receptor signaling	2,514E-03	JAK1, p70 S6 kinase2, H-Ras, MEK2(MAP2K2)
44	Development_Negative regulation of WNT/Beta-catenin signaling in the cytoplasm	2,678E-03	ELAVL1 (HuR), DAB2, Tcf(Lef), Axin, YAP1/TAZ, YAP1 (YAp65)
45	Androgen receptor activation and downstream signaling in Prostate cancer	2,681E-03	JAK1, GCR, NCOA3 (pCIP/SRC3), H-Ras, MEK2(MAP2K2), FEN1, Versican
46	Role of growth factor receptors transactivation by Hyaluronic acid / CD44 signaling in tumor progression	2,802E-03	CD44, MEK1/2, H-Ras, MDR1
47	Signal transduction_IGF-1 receptor signaling pathway	2,973E-03	JAK1, FKHR, 4E-BP1, MEK1/2, H-Ras
48	Cytoskeleton remodeling_Keratin filaments	3,110E-03	Tubulin beta, BPAG1, Tubulin alpha, Tubulin (in microtubules)
49	IGF-1 signaling in pancreatic cancer	3,110E-03	JAK1, H-Ras, NUAK1, MEK2(MAP2K2)
50	WNT signaling in gastric cancer	3,110E-03	CD44, Tcf(Lef), Axin, TCF7L2 (TCF4)
